# Acceptability and usability of home-based HIV and blood glucose self-testing and home-based blood pressure measurement in Kenya, South Africa, and Zambia

**DOI:** 10.1371/journal.pgph.0005445

**Published:** 2026-09-25

**Authors:** Carolyn R. Oliver, Meagan J. Bemer, Amber Lauff, Jennifer F. Morton, Shawna Cooper, Hilton Humphries, Anjali Sharma, Derek Pollard, Anna Winters, Alastair van Heerden, Elizabeth Bukusi, Dino Rech, Paul K. Drain

**Affiliations:** 1 University of Washington School of Medicine, Seattle, Washington, United States of America; 2 Department of Global Health, University of Washington, Seattle, Washington, United States of America; 3 Audere, Seattle, Washington, United States of America; 4 Audere, Johannesburg, South Africa; 5 Human Sciences Research Council, Pretoria, South Africa; 6 Centre for Infectious Disease Research in Zambia, Lusaka, Zambia; 7 Akros Research, Lusaka, Zambia; 8 Akros Research, Missoula, Montana, United States of America; 9 SAMRC/WITS Developmental Pathways for Health Research Unit, Department of Paediatrics, School of Clinical Medicine, Faculty of Health Sciences, University of the Witwatersrand, Johannesburg, Gauteng, South Africa; 10 Center for Microbiology Research, Kenya Medical Research Institute, Nairobi, Kenya; 11 Vanderbilt University Medical Centre, Nashville, Tennessee, United States of America; 12 Department of Epidemiology, University of Washington, Seattle, Washington, United States of America; PLOS: Public Library of Science, UNITED STATES OF AMERICA

## Abstract

Home-based self-testing may improve individual health outcomes and public health programs by lowering barriers associated with clinic-based testing in low- and middle-income countries (LMICs). We assessed the acceptability and usability of conducting home-based self-testing for HIV and blood glucose and home-based researcher-conducted testing for blood pressure in sub-Saharan Africa. We enrolled participants (≥15 years old) from households in peri-urban and rural communities of Kenya, South Africa, and Zambia. Participants opted in to self-directed testing for HIV and blood glucose and had their blood pressure measured by a research team member. Our primary measures included HIV status and testing history, HIV and blood glucose test results, blood pressure, self-reported usability and acceptability of self-testing, and participant preferences for future self-testing. Among the 526 participants from 100 households enrolled per country, the average age was 41 years and 63% were female. Overall, 16% of participants reported living with HIV. Over half of participants (52%) had last tested for HIV > 12 months ago or had never tested for HIV, and 8% of participants were unsure of their HIV status. Among participants who self-tested, 2% (n = 6/330) tested positive for HIV and 4% (n = 18/469) had high blood glucose, while 26% (n = 131/502) had high blood pressure measured by the study team. Only 14% of study participants reported previous self-testing. Most (>90%) participants who self-tested rated all procedures for HIV and blood glucose tests as either “very easy” or “fairly easy” to use. Most participants (87%, n = 458/526) preferred home-based testing. Home-based self-testing for HIV and blood glucose and home-based blood pressure measurement were acceptable, usable and preferred in peri-urban and rural areas of Kenya and South Africa; findings from Zambia suggest promise for home-based testing but were limited by staff-administered HIV tests. Self-testing has the potential to expand and accelerate access to healthcare delivery in LMICs.

## Introduction

In low- and middle-income countries (LMICs), the high burden of acute infections [1] (e.g., HIV, TB, malaria, COVID-19) and the growing prevalence of non-communicable diseases (NCDs) [2] (e.g., diabetes, hypertension, heart disease) pose challenges for healthcare delivery [3,4]. Barriers to clinic-based testing include cost of transportation, long wait times, lost results, inadequate clinic staffing, lack of consistently available diagnostic test stock, and perceived stigma [5–9]. At-home testing, especially when self-administered, offers a promising alternative to clinic and hospital-based care by increasing privacy and encouraging earlier diagnostic testing, which can reduce time to seek and receive treatment – with particularly encouraging developments in HIV diagnosis and treatment [10–12]. Advances in diagnostics, like rapid tests, some of which are approved for self-testing, can expand and accelerate access to healthcare delivery, while empowering individuals with self-care, especially in areas of high prevalence for that condition.

In 2024, the World Health Organization (WHO) published guidelines [13] to advance self-care programs that lower healthcare costs and increase access to care by prioritizing individual agency and self-determination. Self-testing using rapid tests was proposed as one of several healthcare tools that patients could use effectively for self-care. Self-testing offers population health benefits, such as expanding access to healthcare services for individuals who face barriers to clinic-based care [14,15]. Self-testing studies have demonstrated that individuals without medical training can reliably conduct and interpret their own rapid test results [16,17] including in LMICs, albeit primarily demonstrated in single-site studies for a single communicable disease [10,18–23].

We assessed the acceptability and usability of conducting home-based testing with self-administered and researcher-administered tests for communicable diseases and NCDs in multiple peri-urban and rural communities of sub-Saharan Africa, and estimated the presence of HIV, high and low blood glucose, and high blood pressure at a single time point in our study population.

## Materials and methods

### Study design, sites and partners

The DASH (Diagnostic Access to Self-Care & Health Services) Study was a cross-sectional study evaluating the acceptability and usability of home-based testing via household survey and provision of self-testing tools across varied implementation contexts in Kenya, South Africa, and Zambia. Peri-urban communities with populations of 200–2,000 people were chosen within each study site, which included Migori County, Kenya; KwaZulu-Natal Province, South Africa; and Luanshya District, Copperbelt Province, Zambia.

Migori County, Kenya is a peri-urban county of 1.1 million people and faces high burdens of HIV and malaria. Umsunduzi sub-district in KwaZulu-Natal province, South Africa, has approximately 618,000 people living in urban, peri-urban, and rural areas and has a high burden of HIV, sexually transmitted infections (STIs), early pregnancy, and diabetes. Luanshya District, Zambia has 118,000 people and includes peri-urban and rural areas with a high burden of HIV, malaria, and early pregnancy.

The DASH Study used a mobile app, HealthPulse TestNow^TM^ (Audere; Seattle, USA) to guide study participants as they self-tested for HIV and blood glucose. The app was tailored to each study site, accounting for the types and brands of rapid tests used and the local language.

### Eligibility and ethics approvals

Household surveys were conducted by research team members in each of the study sites. Participants were considered members of a household if they spent at least one night there in the past four weeks.

Participants aged 6 months and older were eligible to be enrolled at all study sites. This analysis focused on adolescents and adults aged ≥15 years old. Individuals 16 years and older provided informed written consent to participate, while individuals 15 years of age were required to provide parental consent to participate in the study. The research team collected survey and testing data on encrypted tablet devices using REDCap Version 14. The study was reviewed by the University of Washington Institutional Review Board, the Human Sciences Research Council (HSRC) Research Ethics Committee in South Africa, the Scientific and Ethics Review Unit at the Kenya Medical Research Institute, and ERES Converge IRB in Zambia.

### Assessing acceptability of home-based self-testing

A household survey was conducted that included socio-demographics, current medical conditions, and health behaviors and preferences. Acceptability for home-based testing and self-testing was assessed via survey questions including participants’ preferences for location of future self-testing, preferences for type of self-test, willingness to conduct home-based tests on minors, and willingness to share results of future self-tests with health care workers and public health agencies.

### Assessing usability of home-based self-testing

After conducting the household survey, all participants were offered a mobile app-facilitated test for HIV and blood glucose. The AI-powered app, HealthPulse TestNow, was available on a study-provided mobile device. The app guided participants through the steps of accurately administering and interpreting each test. For participants who opted to self-test, research team members were not allowed to help or guide study participants through self-administering any part of the test. The full version of the app included process control timers, the ability to capture a quality photo of the test, result interpretation guidance, and an artificial intelligence algorithm which runs directly on the mobile device and can interpret results from images of the test. For this study, AI interpreted results were not shared with participants. Participants in Kenya had access to the full version of the app, allowing them to take photos of their test result. In Zambia and South Africa, a prototype of the app was used, and participants did not take photos of their test result. Instead, the test result image was recorded with a photo on a tablet by a research team member.

**Fig 1:**
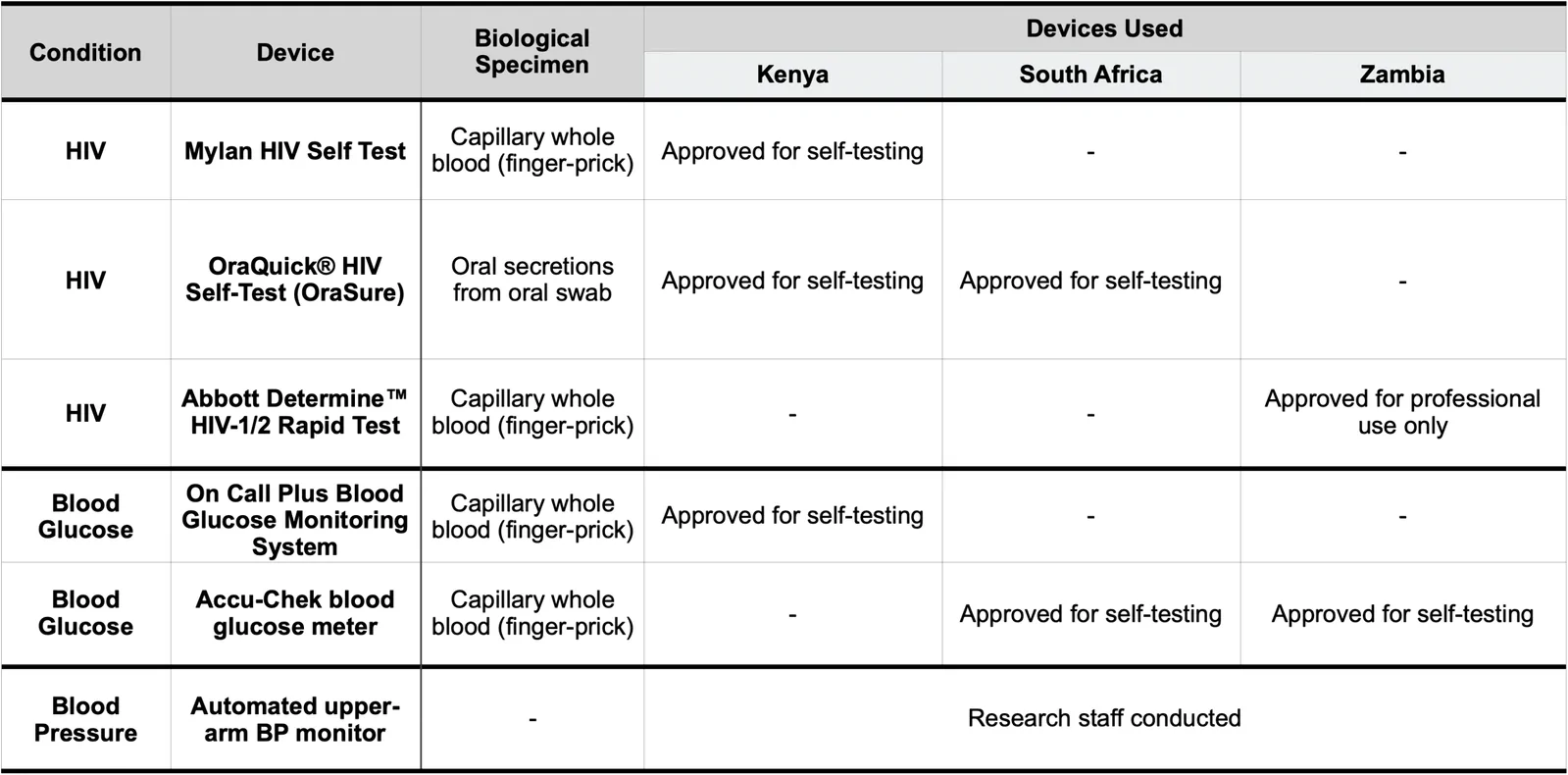
Diagnostic devices, specimen types, and study site utilization. HIV and blood glucose were self-tested in select sites as indicated. In Zambia, HIV testing was staff-administered with participant self-interpretation. Blood pressure measurements were conducted by trained research staff at all sites.

For HIV and blood glucose testing, sites chose rapid tests based on availability and country-level approval for self-testing (Fig 1). The Kenya site used Mylan HIV finger prick self-tests, OraQuick oral HIV self-tests, and the On Call Plus glucose self-testing system. The South Africa site used OraQuick oral HIV self-tests and the Accu-Chek glucose self-testing system. The Zambia site used Abbott Determine finger prick HIV tests and the Accu-Chek glucose self-testing system.

Participants at the Zambia site did not conduct their own HIV test due to stockouts of the OraQuick oral HIV tests which are approved for self-use in Zambia. Instead, a research team member conducted the test using the Abbott Determine finger prick HIV test, approved only for use by trained health workers at the time. Zambian participants were given the study mobile device to follow along in the app and were surveyed only on the ease of understanding the test instructions and were not asked about the ease of collecting a specimen or applying a specimen to the test.

To assess usability, participants who self-tested for HIV and/or blood glucose were asked about the ease of use for four steps in the testing process for each test that they conducted, choosing between “Very easy”, “Fairly easy”, “Fairly difficult”, “Very difficult”, and “Did not attempt”. The four steps were: understanding specimen collection instructions, collecting the specimen, understanding specimen application instructions, and applying the specimen to the test. Participants at all study sites, whether they self-tested or not, were asked to interpret the result of the HIV rapid test and report their interpretation to the research team. The research team, who were all trained to accurately interpret the HIV tests, also interpreted the test result themselves and recorded the result. Participants were asked about the ease of matching their test results to the options provided in the testing instructions and confidence in their stated interpretation of the test.

Participants had the option to have their blood pressure measured by a member of the research team. Digital blood pressure measurement devices were used at each study site, including Omron in Kenya, Contec and Axcess in South Africa, and Citizen in Zambia. Blood pressure measurements were taken with participants seated with both feet on the ground. A single reading was measured and recorded by a member of the research team. Blood pressure self-testing was not included in the study protocol because accurate measurement requires standardized positioning, cuff sizing, and repeated measurements, and the study was not designed to train participants to independently perform and validate these steps.

### Referral to care

In Kenya and South Africa, participants with a positive HIV test were referred for confirmatory testing and care. In Zambia, confirmatory testing was done in the participant’s household and if the confirmatory test was positive, they were referred to care. At all study sites, participants with high or low blood glucose and participants with a high blood pressure measurement were offered referral to care. Study staff provided linkage to care where needed, including transportation support.

### Sample size and statistical analyses

Sample size at each study site was not powered for statistical significance because the primary measures were acceptability and usability. Each study site had a cutoff of 100 households. In Kenya and Zambia, households were selected by convenience sample and the research teams enrolled as many household members as were able and willing to consent. In South Africa, the research team used the Kish grid method [24] to systematically select a household for participation when more than one household was found at a visiting point, and to randomly select a maximum of three members per household. The process involved creating two separate grids -- one with randomly selected visiting points and the other with the number of members to be selected within a household. The Kish grid method ensures a representative and unbiased sample when conducting surveys or studies across various households.

Descriptive statistics were calculated for all study measures, including percentage, mean, standard deviation, and median with interquartile range using Python 3.8.3 and the following packages: pandas, seaborn, scipy, numpy. Participants 15 years and older who participated in any part of the household survey or testing were included in the total study population.

A generalized estimating equation (GEE) logistic regression model with an exchangeable working correlation structure was fit using the statsmodels package in Python 3.8.3 to estimate participants’ preference for home-based future self-testing per study site and overall. This estimate accounts for potential correlation in responses among participants within the same household which could result from privacy concerns, space, and household attitudes towards self-testing.

Missing values for participants were coded accurately as “Unsure/Don’t know,” or “Prefers not to answer.” When participants did not opt-in to self-testing for HIV and/or blood glucose or to blood pressure testing by a research team member, their reason was recorded and reported as such in the Results section. Outlier exclusion was not performed because analyses focused on acceptability and usability outcomes rather than estimation of central tendency or clinical effect sizes, and all observed values were therefore retained.

## Results

The research teams conducted 300 household visits, with 100 households at each study site, between 31 May and 4 August 2023, enrolling 526 adolescent and adult participants (15 years and older) in Kenya (n = 197), South Africa (n = 148), and Zambia (n = 181) (Table 1). An additional 70 participants <15 years old were enrolled in a separate arm of the DASH study but are not included in this analysis as only participants 15 years and older were offered self-testing and surveyed about their testing preferences. The household composition, including enrolled household members *and* those who did not participate in the study, was variable across study sites. In Kenya, households had 5 members on average, while South Africa and Zambia had an average of 2 and 3 household members, respectively. The Kenya site households had an even number of male and female members (48% female) while South Africa and Zambia had majority female participants at 62% and 65%, respectively. Occupation of participants varied across study sites. In Kenya and Zambia, participants primarily reported farming/agriculture, manual labor, and small-market sales or trade occupations. South Africa and Zambia reported high rates of unemployment, 72% and 29%, respectively.

**Table 1:** Demographics of study population.

	Kenya	South Africa	Zambia	All
Households enrolled in survey	100	100	100	300
Number of household members* (Median, IQR)	5 (4,7)	2 (2,3)	3 (2,4)	3 (2,5)
Household Gender Composition (% Female residents) (mean, SD)*	48% (±20%)	62% (±34%)	65% (±31%)	58% (±30%)
Individuals enrolled in survey	197	148	181	526
Female sex	103 (52)	97 (66)	132 (73)	332 (63)
Age, mean (SD)	37 (14)	42 (19)	44 (19)	41 (18)
Occupation				
*Farming/Agriculture*	47 (24)	0	35 (19)	82 (16)
*Housewife*	9 (5)	7 (5)	3 (2)	19 (4)
*Manufacturing/Factory*	0	4 (3)	2 (1)	6 (1)
*Office or Clerical Work*	1 (1)	0	0	1 (0)
*Other Manual Labor*	47 (24)	5 (3)	29 (16)	81 (15)
*Small-market sales or trade*	45 (23)	1 (1)	41 (23)	87 (17)
*Student*	19 (10)	17 (11)	16 (9)	52 (10)
*Unemployed*	8 (4)	106 (72)	53 (29)	167 (32)
*Other*	21 (11)	8 (5)	2 (1)	31 (6)

*Individuals who participated in the study as well as household members who did not participate in the study

**Unless otherwise indicated, values are represented as N (%). Due to rounding, some percentages may not add to 100.

**Fig 2:**
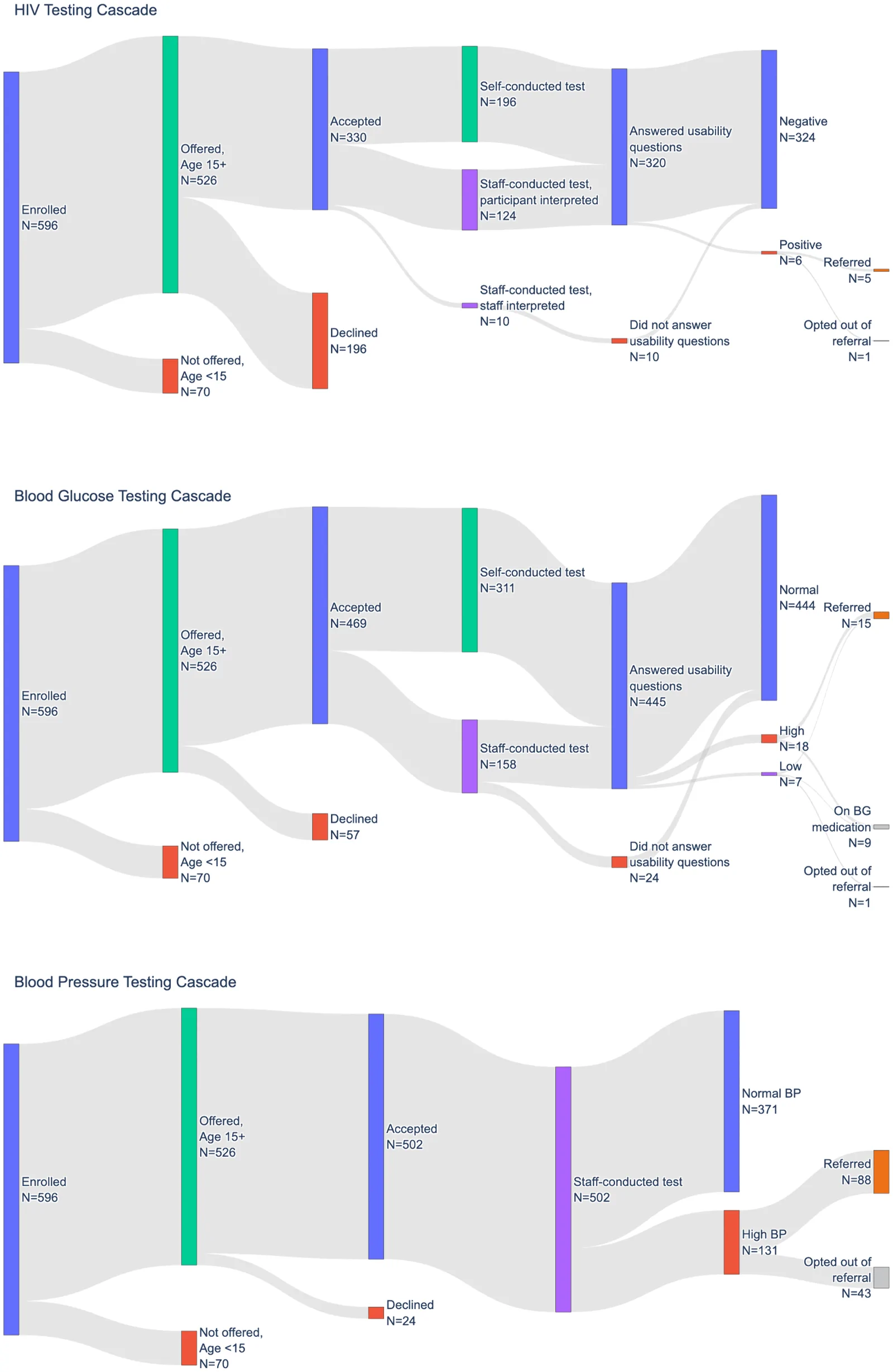
Implementation cascade for testing. (a) HIV testing, (b) Blood glucose testing, (c) Blood pressure measurement. Reasons for declining testing are listed with corresponding Ns in Table 4. Cases in which participants did not complete all steps of the test independently are categorized as “Staff-conducted test.” For any participant with a “Staff-conducted test,” participants were still asked to rate ease of instructions for HIV and blood glucose tests and to interpret the HIV tests and thus are included in “Answered usability questions”.

Participant flow through each stage of the study, including enrollment, testing, answering usability questions, results and referral to care, is presented separately for HIV, blood glucose, and blood pressure testing in Fig 2.

### Usability of self-administered rapid diagnostic tests

**Fig 3:**
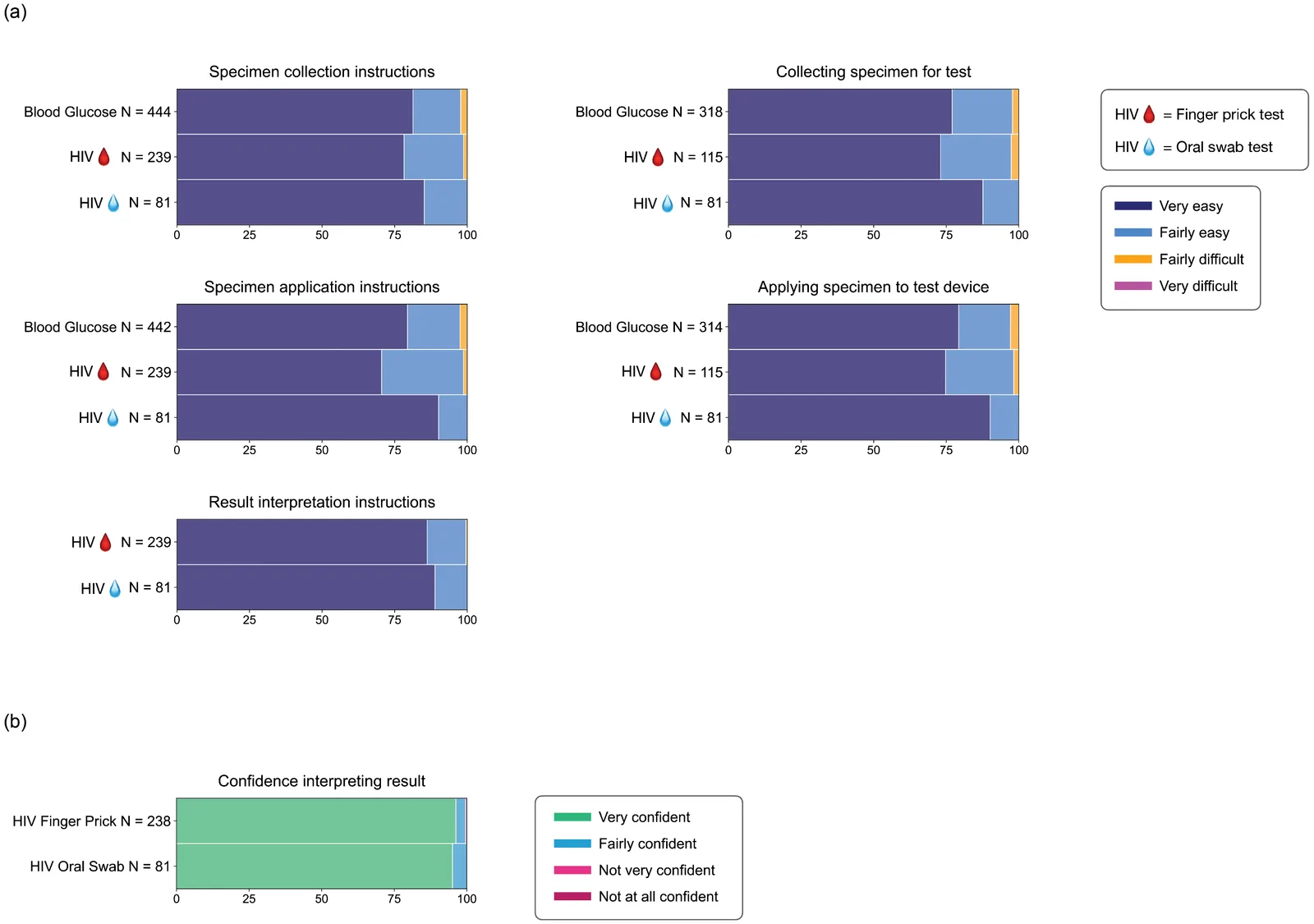
Usability of HIV and blood glucose self-tests. (a) Ease of understanding test instructions or completing test, (b) Confidence interpreting HIV test. Participants in Zambia were not allowed to conduct HIV self-tests due to device regulation and therefore, only answered usability questions about understanding instructions and interpreting test results. Missing responses or the responses ‘Did not attempt to do this' or ‘Did not read the instructions’ are not included in these plots. Exact Ns for each of the responses represented in this figure, stratified by study site, can be found in S1 Table and S2 Table.

Prior experience using a rapid test at home was low — 14% across all sites (Table 2). Despite limited experience with rapid tests, more than 90% of participants across all study sites reported that the app-based instructions for HIV and blood glucose testing were “very easy” or “fairly easy” to understand. Over 90% of participants from Kenya and South Africa who self-tested, found the HIV oral swab, HIV finger prick and blood glucose finger prick tests were “very easy” or “fairly easy” to conduct themselves (Fig 3a, S1 Table, S2 Table). Participants from Zambia were precluded from self-testing for HIV and so did not answer usability questions for collecting and applying the specimen to the HIV test (S1 Table), however reported high ease of understanding HIV testing instructions, similar to the findings in Kenya and South Africa. While 90% of Zambia participants rated the self-testing instructions for blood glucose as “very easy” and “fairly easy,” most participants from Zambia opted not to conduct the self-test for blood glucose themselves (S2 Table). All participants who self-tested for HIV or who were tested for HIV by the research team, using either finger prick or oral swab tests, accurately interpreted their test results, and over 95% of participants reported that they were “very confident” or “fairly confident” with their interpretation (Fig 3b, S1 Table).

**Table 2:** Acceptability of and preferences for any type of rapid test.

	Kenya N = 197	South Africa N = 148	Zambia N = 181	All N = 526
Self-tested with a rapid test prior to the study*	34 (17)	32 (22)	7 (4)	73 (14)
Preference for where to self-test with a rapid test in the future^↑^				
*I would prefer to do this at home*	168 (85)	141 (95)	149 (82)	458 (87)
*I would prefer to do this in a clinical setting (e.g., at a local health clinic or hospital)*	15 (8)	1 (1)	30 (17)	46 (9)
*No preference/Anywhere*	12 (6)	1 (1)	0	13 (2)
*Unsure / Don’t know*	0	0	2 (1)	2 (<1)
Willing to use finger prick test on a 5–11 year old child?^↑^				
*Yes*	147 (75)	60 (41)	131 (72)	338 (64)
*Some children but not all children*	21 (11)	20 (14)	9 (5)	50 (10)
*No*	22 (11)	7 (5)	37 (20)	66 (13)
*Unsure / don’t know*	5 (3)	56 (38)	4 (2)	65 (12)
Willing to use a finger prick test on a 12–15 year old child?^↑^				
*Yes*	170 (86)	75 (51)	134 (74)	379 (72)
*Some children but not all children*	7 (4)	14 (9)	7 (4)	28 (5)
*No*	13 (7)	2 (1)	36 (20)	51 (10)
*Unsure / don’t know*	5 (3)	52 (35)	4 (2)	61 (12)
Willing to use this app or similar to take photos of test result and share with a community health worker in the future?	192 (97)	127 (86)	177 (99)	496 (94)
Willing to use this app or similar to take photos of test result and share with the Ministry of Health in the future?	193 (98)	116 (78)	175 (98)	484 (92)

* Unless otherwise indicated, values are represented as N (%). Due to rounding, some percentages may not add to 100.

↑ Responses were not obtained from 2 participants in Kenya and 5 participants in South Africa, thus total percentages for questions in these study sites may not add to 100.

### Acceptability of self-administered rapid diagnostic tests

When asked about their preferences for diagnostic testing and disease monitoring, most participants preferred home-based testing (87%) over clinic-based testing (9%) (Table 2). When accounting for possible clustering effects within households using a GEE model, estimates for participants’ preference for home-based testing were similar, 85% (95% CI 79–90%) in Kenya, 95% (95% CI: 91–98%) in South Africa, and 81% (95% CI: 74–87%) in Zambia, with an overall preference estimate of 87% (95% CI 84–90%) for home-based future self-testing.

**Fig 4:**
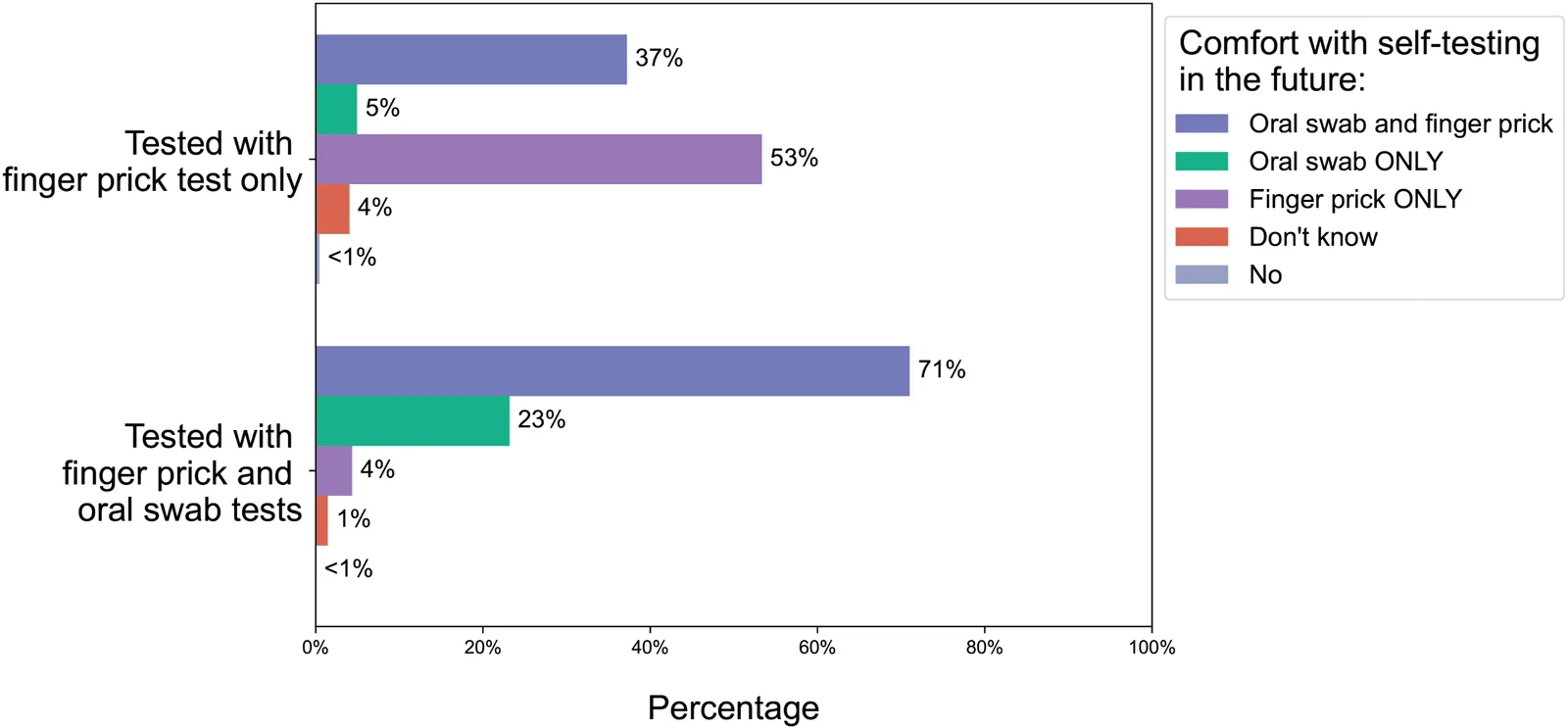
Preference for future self-test type, based on test type used.

Of participants who used an HIV oral swab test *and* a blood glucose finger prick test, 71% said they would be comfortable using either type of test (oral swab or finger prick) on their own in the future, whereas 23% reported they would only be comfortable using an oral swab test on their own in the future (Fig 4). For participants who used finger prick tests for both HIV and blood glucose tests, 37% reported they would be comfortable using either type of test (oral swab or finger prick) on their own in the future, with 53% of participants reporting they would only be comfortable with using a finger prick test in the future. Fewer than 5% of participants in the study population reported they would not be comfortable or were unsure if they would be comfortable conducting a finger prick or oral swab test on their own in the future.

Participants also reported a willingness to share self-test results in the future via a mobile app with community health workers (94%) and their respective Department/Ministry of Health (92%; Table 2).

### Prior HIV testing and HIV prevalence

The proportion of participants who were aware of living with HIV prior to study start varied across the study sites, with 14% in Kenya, 25% in South Africa, and 10% in Zambia (Table 3). Among participants who did not report a positive HIV status prior to study enrollment, 5% in Kenya, 4% in South Africa, and 15% in Zambia had never been tested for HIV and 39% in Kenya, 50% in South Africa, and 46% in Zambia had not been tested for HIV in over 12 months. For all participants without a known positive HIV status prior to the study, 68% in Kenya, 45% in South Africa, and 72% in Zambia consented to test for HIV, and between 1–3% of those participants received a positive HIV rapid test and were referred for confirmatory testing (Table 4). Of the 37% (n = 196) of participants who did not test for HIV in this study, 42% (n = 83) had a previous seropositive HIV result and were not eligible to test for HIV. Of the remaining 58% of the participants (n = 113; 21% of the total study population), one participant did not test in South Africa due to test stockout and 112 other participants across study sites declined to test for HIV and did not report a previous seropositive HIV result (Table 4).

**Table 3:** HIV status and testing history and estimated national prevalence of HIV, type 2 diabetes, and hypertension.

	Kenya N = 197	South Africa N = 148	Zambia N = 181	All N = 526
Self-reported HIV status prior to self-testing*				
*Positive*	28 (14)	37 (25)	18 (10)	83 (16)
*Negative*	154 (78)	88 (59)	134 (74)	376 (71)
*Unsure/ doesn’t know*	15 (8)	14 (9)	27 (15)	56 (11)
*Prefers not to answer*	0	9 (6)	2 (1)	11 (2)
Time Since Last HIV Test				
*< 2 weeks*	11/169 (7)	1/111 (1)	2/163 (1)	14/443 (3)
*2 weeks -2 months*	16/169 (9)	0	12/163 (7)	28/443 (6)
*2–5 months*	41/169 (24)	8/111 (7)	16/163 (10)	65/443 (15)
*6–12 months*	21/169 (12)	23/111 (21)	17/163 (10)	61/443 (14)
*> 12 months*	66/169 (39)	55/111 (50)	75/163 (46)	196/443 (44)
*Has never had a HIV test*	8/169 (5)	4/111 (4)	25/163 (15)	37/443 (8)
*Unsure / doesn’t know*	6/169 (4)	13/111 (12)	15/163 (9)	34/443 (8)
*Prefers not to answer*	0	7/111 (6)	1/163 (1)	8/443 (2)
Estimated National Prevalence of HIV, Type 2 Diabetes, and Hypertension				
	Kenya	South Africa	Zambia	
HIV Prevalence, estimated %				
*Female, 15–49*	5.2 [25]	22.3 [26]	14.2 [27]	
*Male, 15–49*	4.5 [25]	11.0 [26]	7.5 [27]	
Type 2 Diabetes Prevalence, estimated %	2.4 [28]	15.3 [29] ^∬^	3.5 [30] ^∫^	
Hypertension^↟^ Prevalence, estimated %	24.5 [31]	44, 46 [32] ^∭^	25.9 [33]	

* Unless otherwise indicated, values are represented as N (%). Due to rounding, some percentages may not add to 100.

↟ Defined as systolic pressure ≥ 140mmHg or diastolic pressure ≧ 90mmHg or taking medication for hypertension

∫Defined as random blood glucose ≥ 11.1 mmol/L or self-reported diabetes diagnosis

∬Defined as fasting plasma glucose (FPG) ≥ 7.0 mmol/L, 2-h oral glucose tolerance test (OGTT) plasma glucose ≥ 11.1 mmol/L, glycated hemoglobin (HbA1c) ≥ 6.5% (48 mmol/mol), or self-reported use of diabetes drugs.

∭Hypertension prevalence among males and females respectively.

**Table 4:** Results of rapid diagnostic tests for HIV, blood glucose and blood pressure.

	Kenya N = 197	South Africa N = 148	Zambia N = 181	All N = 526
Tested for HIV*	133 (68)	66 (45)	131 (72)	330 (63)
*Negative*	129/133 (97)	65/66 (99)	130/131 (99)	324/330 (98)
*Positive*	4/133 (3)	1/66 (2)	1/131 (1)	6/330 (2)
Did not test for HIV because…	64/197 (32)	82/148 (55)	50/181 (28)	196/526 (37)
*participant previously tested positive for HIV*	28/64 (44)	37/82 (45)	18/50 (36)	83/196 (42)
*participant declined*	36/64 (56)	44/82 (54)	32/50 (64)	112/196 (57)
*test stockouts*	0	1/82 (1)	0	1/196 (1)
Tested blood glucose^↑^	177 (90)	136 (92)	156 (86)	469 (89)
Blood glucose (mmol/L) (mean, SD)	6.1 (2.6)	6.6 (2.0)	6.2 (1.5)	6.3 (2.1)
Low blood glucose (≤3.9 mmol/L)	3/177 (2)	2/136 (2)	2/156 (1)	7/469 (1)
High blood glucose (> 10 mmol/L)	5/177 (3)	8/136 (6)	5/156 (3)	18/469 (4)
Did not test blood glucose because…	20 (10)	12 (8)	25 (14)	57 (11)
*participant declined*	20/20 (100)	10/12 (83)	21/25 (84)	51/57 (89)
*could not get enough blood from finger prick*	0	0	4/25 (16)	4/57 (7)
*research team ran out of test/ materials*	0	1/12 (8)	0	1/57 (2)
*other reason*	0	1/12 (8)	0	1/57 (2)
Tested blood pressure ^↑^	196 (99)	133 (90)	173 (96)	502 (95)
Systolic BP (mmHg) (mean, SD)	118.8 (17)	133.3 (20)	134.0 (29)	127.9 (24)
Diastolic BP (mmHg) (mean, SD)	76.8 (10)	79.6 (12)	85.6 (15)	80.6 (13)
High BP (systolic ≥ 140mmHg or diastolic ≥ 90mmHg)	24/196 (12)	38/133 (29)	69/173 (40)	131/502 (26)

* Unless otherwise indicated, values are represented as N (%). Due to rounding, some percentages may not add to 100.

↑ Participants tested for blood glucose during household visits and had not necessarily fasted before the blood glucose test. Blood pressure was measured at a single time point.

### Blood glucose and blood pressure testing results

Over 85% of participants at all study sites consented to non-fasting blood glucose testing (Table 4). Between 3%-6% of participants had high blood glucose readings (> 10 mmol/L) and between 1%-2% of participants had low blood glucose readings (≤ 3.9 mmol/L). Greater than 90% of participants across study sites consented to having their blood pressure measured by a member of the research team. Prevalence of high blood pressure varied across sites, with 12% in Kenya, 29% in South Africa and 40% in Zambia (Table 4).

## Discussion

In this cohort of adolescents and adults in peri-urban and rural areas of Kenya, South Africa, and Zambia, home-based, self-testing was often an acceptable, usable, and preferred method to test for HIV and measure blood glucose. Despite few participants having experience with self-testing using a rapid test, most participants in Kenya and South Africa reported that home-based self-testing was usable and acceptable for both finger prick blood and oral swab specimen collection. While the research team was not allowed to assist participants who opted to self-test, it is possible that their presence at this initial self-testing informed participants’ reported confidence for future self-testing. Most participants in Zambia reported that home-based self-testing *instructions* were usable for blood glucose testing but provided feedback to the study team that lack of familiarity with diabetes and blood glucose testing deterred them from attempting to self-test. Zambian participants reported to study staff that the test was new to them and more technical than other self-tests they were familiar with, e.g., malaria. They reported that they would want assistance from a community health worker for the first time before attempting to conduct the test themselves. They also reported concern about handling the blood glucometer because it looked expensive. This finding highlighted a distinct difference between study sites in participants’ willingness to attempt a novel self-test. Participants across all sites overwhelmingly reported a preference to conduct self-testing at home rather than self-testing in a clinic-setting and an openness to report their future self-testing results to public health agencies. Participants who conducted self-tests using both oral swab and finger prick test types reported a greater comfort using either test type in the future, indicating that even a single exposure to self-administered test types may increase individual participation in subsequent self-testing programs. Participants who were given only finger prick tests were more likely to report comfort using only finger prick tests in the future. Gaining experience using oral swab tests seems to increase an individual’s comfort using that test type on their own in the future. This finding suggests that larger home-based self-testing programs, which may struggle with rapid test availability, can implement programs by pooling multiple test types. Across study sites, 63% of participants tested for HIV, 16% did not test because of known seropositivity for HIV, and the remaining 21% opted not to test, some giving no reason and others stating that they had tested for HIV recently and preferred not to test again. Finally, most participants in our study found blood pressure measurement conducted by a trained research staff member to be an acceptable home-based test.

While our study is the first to evaluate acceptability and usability of home-based testing to screen for communicable *and* non-communicable diseases across multiple country study sites, our findings are consistent with HIV home-based self-testing studies and meta-analyses that have demonstrated a high degree of usability for rapid self-testing, an acceptability of home-based testing over clinic-based testing, and accurate test result interpretation by participants [11,16,22,34,35]. Our study participants’ high rate of confidence in HIV result interpretation was similarly found in another HIV self-testing study in South Africa [11]. Previous studies offer mixed results for preference between HIV finger prick tests versus HIV oral swab tests, when given the option [36,37]. Our study found that when participants were given the opportunity to use both test types, 69% were comfortable using either test type in the future. While this study did not measure participants’ success of or preferences for linkage-to-care following a positive HIV self-test result, our research team did refer those individuals to follow-up testing and care, a similar study design to other HIV self-testing studies [11,34]. We found that most participants were willing to share their test results with a community health worker and their respective public health agency. A prior HIV self-testing study in Uganda [38] examined willingness to disclose results to family members or partners, however, we did not identify comparable literature examining willingness to share self-test results for HIV or blood glucose with community health workers or government health authorities, warranting future investigation to compare to our finding.

We confirmed that individuals find home-based blood glucose testing acceptable across all sites, however there was variability between sites in terms of willingness to self-test. Most participants in Kenya and South Africa completed blood glucose self-testing, whereas most Zambia participants opted out of self-testing due to lack of familiarity with tests and fear of using what was perceived as expensive equipment. Studies of glycemic control in patients diagnosed with type 2 diabetes in Zambia indicate that self-monitoring with glucometers is rare [39]. The preference for home-based testing and high degree of usability of self-testing for blood glucose in Kenya and South Africa is consistent with other studies evaluating self-monitored blood glucose (SMBG) programs in LMICs [40,41]. We anticipate that core usability findings, such as self-testing device and instruction comprehension and comfort, are likely transferable across similar LMIC settings, whereas site-specific factors, including stockouts and equipment familiarity, indicate that local supply chains and prior exposure to testing technologies are important factors that could impede future home-based self-testing programs. Future research is warranted to investigate the usability of blood glucose self-tests at home in Zambia.

Our study sites represent the regional variability in HIV, high blood glucose, and high blood pressure prevalence within each of their respective countries. Each home-based test used in this study is a screening tool requiring further diagnostic testing, and thus our HIV positivity, high and low blood glucose, and high blood pressure findings cannot be compared to national estimates of HIV seropositivity, diabetes and hypertension respectively. However, we present those national estimates in Table 3 to contextualize the underlying burden of disease and the potential need for point-of-care HIV, blood glucose and blood pressure testing. We observed similar point-in-time HIV positivity in study participants at the South Africa (26%) and Zambia (10%) sites when compared to national estimates of HIV seropositivity. In Kenya, 16% of participants either had a positive HIV test or had previously screened positive for HIV, higher than HIV seropositivity national estimates of 5.2% for females and 4.5% for males but consistent with prevalence in the sampled Migori County (Table 3).

We found a similar prevalence of high blood glucose among study participants in Kenya (3%) and Zambia (3%) compared to national estimates for diabetes. In South Africa, 6% of participants’ tests met the threshold for high blood glucose whereas national estimate of diabetes is 15.3% (Table 3).

The percentage of participants with high blood pressure readings was 12%, 29% and 40% in Kenya, South Africa and Zambia respectively. The national estimates for hypertension in Kenya and Zambia are 25% and 26% respectively. South Africa national estimates are reported as 44% male and 46% female (Table 3). While single time point blood pressure readings are not diagnostic of hypertension, we include the national prevalence estimates to demonstrate the need for longitudinal blood pressure measurements in these study countries. Our findings indicate that HIV, high blood glucose, and high blood pressure are major health issues in each of these study sites.

HIV self-testing comes with risks including misinterpretation of test results; however, this study’s findings provide reassurance that individuals can accurately interpret seronegative and seropositive HIV results using app-based guidance. Interpreting continuous values like blood glucose and blood pressure, which can fluctuate throughout the day, will require greater patient education, especially given that diabetes and hypertension are both new public health issues and are widely underdiagnosed and undertreated in the study sites [42,43]. However, studies of patient education and disease management programs for diabetes and hypertension self-monitoring in LMICs are encouraging [40,41,44–46].

Addressing the availability and affordability of rapid tests approved for self-use is crucial for the sustainability of future self-testing programs. One of the strengths of our study was eliminating typical barriers to access tests by providing the rapid test and study mobile devices with a digital tool so that we could study acceptability and usability of the tests themselves. However, future self-testing programs seeking to measure the feasibility of such tests will need to take into account logistic and economic challenges facing individuals, including but not limited to test stockouts, expensive measuring tools for blood glucose and blood pressure monitoring, greater required quantity of blood glucose tests vs. HIV tests given testing frequency, and continuity between self-testing and confirmatory diagnosis with follow-up treatment.

The high degree of willingness of participants to test for HIV, blood glucose and blood pressure and to share their results with community health workers and Ministries/Departments of Health signals an opportunity for future research to explore the design and implementation of self-testing public health surveillance programs in LMICs. One possible mechanism for sharing test results with public health agencies and research studies is using an app like the one used in this study, which demonstrated the ability to guide participants through the self-testing process. However, utilizing an app also poses clinical and legal challenges that should be considered for future self-testing programs, like data privacy and the app’s responsibility to correctly interpret the test results.

Our study had several strengths and limitations. Unlike many other acceptability and usability studies which have evaluated rapid tests for a single, communicable disease like HIV, this study evaluated tests for communicable and non-communicable diseases in the same study population. This study also demonstrated the acceptability and usability of multiple types of rapid tests, including finger prick blood and oral swab tests, across multiple study sites in sub-Saharan Africa, indicating that future research and disease monitoring programs can likely rely on pooling test types for efficient and user-friendly self-testing.

An important limitation of this study was the lack of availability of HIV tests that could be used for self-testing in Zambia as previously described, which limited our ability to evaluate the usability of these tests for self-testing at this study site. While participants were still asked to rank the ease of understanding testing instructions, this cannot replace measuring usability of conducting the test themselves. Relatedly, participants in Zambia were largely unwilling to self-test their blood glucose levels, a decision which could have been impacted by participants being prevented from self-testing for HIV. This finding could also represent a study site difference in willingness to try unfamiliar self-tests. Both findings warrant future investigation into acceptability and usability of HIV and blood glucose self-testing in Zambia.

Due to its cross-sectional nature, this study’s single time point blood pressure testing was not meant to be diagnostic for hypertension. Likewise, the use of blood glucose tests without a fasting protocol was not diagnostic of diabetes. Selection bias introduced by differences in sampling strategies across sites—convenience sampling in Kenya and Zambia versus Kish grid–based random selection in South Africa—may impact findings by restricting both internal consistency and cross-site comparisons.

While participants reported strong usability metrics for all self-administered tests and a high prevalence of comfort sharing future self-test results with either community health workers or a Ministry/Department of Health, this may represent selection bias as our study population had all opted into a self-testing study, indicating they may be less concerned about stigma and privacy. Additionally, the presence of our study staff may have introduced social desirability bias, influencing participants’ survey responses. Future research in which self-administered tests are given to participants to take home with instructions on sharing results with their corresponding Ministry/Department of Health would build on the work of this study and better estimate acceptability and willingness to share results for public health efforts.

## Conclusion

Home-based rapid testing can empower individuals to self-test for communicable and non-communicable diseases. The results of this study indicate that self-testing for HIV and blood glucose is an acceptable and usable method for participants in two of the three sites studied. Self-testing alerts individuals as to whether they need to seek confirmatory testing in a clinic-based setting and, potentially, future treatment. Our findings provide evidence that individuals value home-based self-testing, and support efforts to broaden the application of self-testing to more diseases in LMICs.

## Supporting information

S1 Table Usability of HIV self-tests. (TIF)

S2 Table Usability of blood glucose self-tests. (TIF)

S1 File STROBE Checklist: Checklist of items that should be reported in cross-sectional studies. (DOCX)

S2 File PLOS Inclusivity In Global Research checklist. (PDF)
