# Supplementary material for: Acceptability and usability of home-based HIV and blood glucose self-testing and home-based blood pressure measurement in Kenya, South Africa, and Zambia
Source: PLOS Glob Public Health. 2026 Sep 25;6(9):e0005445. doi: 10.1371/journal.pgph.0005445 (PMC13614574; doi:10.1371/journal.pgph.0005445)
Supplement: Supplementary file 2 [file pgph.0005445.s003.docx]

**STROBE Statement**

*Checklist of items that should be included in reports of cross-sectional studies*

**Manuscript:** Acceptability and Usability of Home-based HIV and Blood Glucose Self-Testing and Home-based Blood Pressure Measurement in Kenya, South Africa, and Zambia

**Authors**: Carolyn Oliver, Meagan J. Bemer, Amber Lauff, Jennifer F. Morton, Shawna Cooper, Hilton Humphries, Anjali Sharma, Derek Pollard, Anna Winters, Alastair van Heerden, Elizabeth Bukusi, Dino Rech, Paul K. Drain

| **Section/Topic** | **Item No** | **Recommendation** | **Reported on page/line #** | **Author notes** |
| --- | --- | --- | --- | --- |
| Title and abstract | 1 | (a) Indicate the study's design with a commonly used term in the title or the abstract  (b) Provide in the abstract an informative and balanced summary of what was done and what was found | Pages 1-2 |  |
| **Introduction** |  |  |  |  |
| Background/rationale | 2 | Explain the scientific background and rationale for the investigation being reported | Pages 3-4 |  |
| Objectives | 3 | State specific objectives, including any prespecified hypotheses | Pages 3-4 |  |
| **Methods** |  |  |  |  |
| Study design | 4 | Present key elements of study design early in the paper | Page 4 |  |
| Setting | 5 | Describe the setting, locations, and relevant dates, including periods of recruitment, exposure, follow-up, and data collection | Page 4 (setting, locations)  Page 9 (dates of recruitment, data collection) |  |
| Participants | 6 | (a) Give the eligibility criteria, and the sources and methods of selection of participants | Pages 4-5 |  |
| Variables | 7 | Clearly define all outcomes, exposures, predictors, potential confounders, and effect modifiers. Give diagnostic criteria, if applicable | Pages 5-7 |  |
| Data sources/measurement | 8* | For each variable of interest, give sources of data and details of methods of assessment (measurement). Describe comparability of assessment methods if there is more than one group | Pages 5-7  Figure 1 (details exact models of rapid tests and study regulations for self-testing) |  |
| Bias | 9 | Describe any efforts to address potential sources of bias | Page 5 (describes how study staff could not assist with any part of self-testing) |  |
| Study size | 10 | Explain how the study size was arrived at | Page 8 |  |
| Quantitative variables | 11 | Explain how quantitative variables were handled in the analyses. If applicable, describe which groupings were chosen and why | Pages 8-9 |  |
| Statistical methods | 12 | (a) Describe all statistical methods, including those used to control for confounding  (b) Describe any methods used to examine subgroups and interactions  (c) Explain how missing data were addressed  (d) If applicable, describe analytical methods taking account of sampling strategy  (e) Describe any sensitivity analyses | Pages 8-9 |  |
| **Results** |  |  |  |  |
| Participants | 13* | (a) Report numbers of individuals at each stage of study—e.g. numbers potentially eligible, examined for eligibility, confirmed eligible, included in the study, completing follow-up, and analysed  (b) Give reasons for non-participation at each stage  (c) Consider use of a flow diagram | Pages 9-11 and Figure 2 (full implementation cascade for participants across each step in the study enrollment, testing, survey and referral) |  |
| Descriptive data | 14* | (a) Give characteristics of study participants (e.g. demographic, clinical, social) and information on exposures and potential confounders  (b) Indicate number of participants with missing data for each variable of interest | Pages 9-10 and Table 1 | Missingness included in each table |
| Outcome data | 15* | Report numbers of outcome events or summary measures | Pages 11-19 |  |
| Main results | 16 | (a) Give unadjusted estimates and, if applicable, confounder-adjusted estimates and their precision (e.g., 95% confidence interval). Make clear which confounders were adjusted for and why they were included  (b) Report category boundaries when continuous variables were categorized  (c) If relevant, consider translating estimates of relative risk into absolute risk for a meaningful time period | Pages 11-19 |  |
| Other analyses | 17 | Report other analyses done—e.g. analyses of subgroups and interactions, and sensitivity analyses | NA |  |
| **Discussion** |  |  |  |  |
| Key results | 18 | Summarise key results with reference to study objectives | Pages 19-20 |  |
| Limitations | 19 | Discuss limitations of the study, taking into account sources of potential bias or imprecision. Discuss both direction and magnitude of any potential bias | Pages 24-25 |  |
| Interpretation | 20 | Give a cautious overall interpretation of results considering objectives, limitations, multiplicity of analyses, results from similar studies, and other relevant evidence | Pages 20-24 |  |
| Generalisability | 21 | Discuss the generalisability (external validity) of the study results | Pages 23-24 |  |
| **Other information** |  |  |  |  |
| Funding | 22 | Give the source of funding and the role of the funders for the present study and, if applicable, for the original study on which the present article is based | Acknowledged in another submission section for the PLOS Global Public Health Journal |  |

** Give information separately for exposed and unexposed groups, where applicable.*

*Note: An Explanation and Elaboration article discusses each checklist item and gives methodological background and published examples of transparent reporting. The STROBE checklist is best used in conjunction with this article, freely available on the Web sites of PLOS Medicine, Annals of Internal Medicine, and Epidemiology. Information on the STROBE Initiative is available at www.strobe-statement.org.*
