# Supplementary material for: Acceptability and usability of home-based HIV and blood glucose self-testing and home-based blood pressure measurement in Kenya, South Africa, and Zambia
Source: PLOS Glob Public Health. 2026 Sep 25;6(9):e0005445. doi: 10.1371/journal.pgph.0005445 (PMC13614574; doi:10.1371/journal.pgph.0005445)
Supplement: Supplementary file 4 [file pgph.0005445.s007.docx]

February 28, 2026

Manuscript Title: “*Feasibility and Preferences for Home-based HIV and Blood Glucose Self-Testing and Home-based Blood Pressure Measurement in Kenya, South Africa, and Zambia*”

Dear Dr. Francis,

Thank you for providing comments and inviting us to resubmit a revised version of our paper. We have provided a point-by-point response to the editorial review below. Reviewer feedback is provided in italics and our responses are listed below each piece of feedback. We have also submitted a clean version of the revised manuscript without the use of Track Changes.

All authors have contributed significantly to the work, have seen and approved of this manuscript, and can attest to the validity and honesty of the results. The contents of this article have not been published, and the paper is not under review elsewhere for publication. Thank you for your consideration.

Please direct all correspondence to:

Carolyn R Oliver, MS

325 Ninth Ave; HMC# 359927; Seattle, WA 98104

Tel: +1 214-300-1777; E-mail: [croliver@uw.edu](mailto:croliver@uw.edu)

Sincerely,

**Carolyn R Oliver, MS**

*Medical Student*

*University of Washington School of Medicine*

Tel: (214)300-1777; [croliver@uw.edu](mailto:croliver@uw.edu)

Thank you for allowing us to make revisions that strengthen the manuscript’s content and fit within the journal's style. Here is a summary of the changes made, based on the list of reviewer feedback received via email.

1. ***Reviewer Feedback:*** *“Zambia did NOT conduct true HIV self-testing (Lines 118 – 122), this is a critical methodological issue, as this is a primary outcome of the study. Participants only self-interpreted the results; they did not self-administer the test. Despite this deviation, Zambia’s results are included in pooled analyses of ‘ease,’ ‘confidence,’ and ‘feasibility.’*

*This undermines the conclusions made as it inflates the usability metrics, biases pooled results upwards and makes acceptability comparisons across sites invalid. It basically challenges the main conclusion of the study that “home-based self-testing for HIV… was feasible across all sites.”*

- *It is important to conduct sensitivity analyses excluding Zambia’s HIV findings, present country-stratified feasibility results, revise the conclusions made, and mainly clarify whether the aim was self-testing or home testing facilitated by staff.”*

**Response:**

We have clarified throughout the manuscript that in Zambia, HIV testing was conducted by trained research staff, with participants reporting only their understanding of the instructions for self-testing and self-interpreting results rather than self-administering the test. To address concerns about bias and comparability, we have revised the Methods to explicitly distinguish HIV self-testing from staff-facilitated testing and presented country-stratified feasibility results in Supplemental Table 1. These analyses are now reported in the Results and referenced in the Discussion. Our conclusions have been revised to distinguish between the feasibility of home-based HIV assisted testing and home-based HIV self-testing across all sites.

In addition, we further clarified limited participant involvement in blood glucose testing in Zambia by expanding the Discussion and adding Supplemental Table 1, which details site-specific testing usability measures and participant participation. These additions describe how HIV self-testing regulations and a lack of familiarity with blood glucose testing in Zambia may have influenced participants in Zambia to opt out of blood glucose self-testing.

2. ***Reviewer Feedback:*** *“Hypertension was not self-tested (Lines 128 n-130), the title and conclusion suggest self-testing of all three conditions, but BP measurements were performed by research staff, not participants. Provide an explicit justification that BP self-testing was excluded due to protocol, device complexity, or concerns about measurement validity. Revise the title and abstract to reflect that hypertension was measured at home by staff, not self-tested, and clarify in the Methods and Discussion how this affects comparability across disease conditions.”*

**Response:**

We have revised the manuscript to clearly reflect that blood pressure was measured in the home by trained research staff rather than self-tested by participants. The title, abstract, and conclusions have been updated accordingly to distinguish self-testing (HIV and blood glucose) from staff-administered, home-based blood pressure measurement. We have also added justification in the Methods noting that blood pressure self-testing was not included due to protocol design and concerns regarding measurement validity and device complexity. The Discussion now explicitly addresses how this difference limits direct comparability across disease conditions.

3. ***Reviewer Feedback:*** *“At Lines 134–141, the manuscript describes differing sampling approaches: Convenience sampling in Kenya and Zambia, and Kish grid random selection in South Africa. These methodological inconsistencies affect internal validity and cross-country comparability, yet their implications are minimally discussed. Please expand the limitations section to explicitly address how non-probability sampling may bias feasibility estimates and prevalence comparisons.”*

**Response:**

We have expanded the Limitations section to explicitly discuss how differences in recruitment strategies—convenience sampling in Kenya and Zambia and household-based random selection in South Africa—may affect internal validity and cross-country interpretation. The revised manuscript clarifies that non-probability sampling is a limitation to comparing feasibility estimates and disease prevalence across sites.

4. ***Reviewer Feedback:*** *Interpretations of Clinical Measurements*

*“In your methods, you clearly state that Blood glucose tests were conducted without fasting (Line 221–222) and blood pressure was taken at a single time point (Line 286–287); however, in the Results section, the values are presented alongside national prevalence estimates (Table 3), which could mislead readers into interpreting your findings as prevalence data. Although the Discussion acknowledges this limitation, it is important to make clear throughout that readings are point in time, not diagnostic, and cannot be compared directly to national prevalence.”*

**Response:**

We have revised the Table 4 caption and relevant Methods, Results and Discussion text to clearly state that blood glucose and blood pressure measurements represent single, non-diagnostic point-in-time readings. References to national prevalence estimates are now explicitly framed as epidemiologic context rather than direct comparators. Additional clarifying language has been added to the Discussion to ensure that readers do not interpret these measurements as diagnostic or prevalence estimates.

5. ***Reviewer Feedback:*** *Feasibility and Usability Findings May Be Influenced by Bias (Lines 165–167; 236–243)*

*“The manuscript reports exceptionally high ease-of-use ratings (≥90%) across all tests (Lines 165–167; 236–243). While encouraging, these findings may reflect: Social desirability bias during household visits, the Hawthorne effect due to the presence of research staff, and variation in test type availability across sites. It is important to expand the Limitations section to discuss how these biases could inflate feasibility ratings.”*

**Response:**

The Limitations section has been expanded to discuss the potential impact of social desirability bias during household visits in the presence of research staff. We have also suggested future research that could further test our findings by sending out self-test kits to be used without the presence of a research team member with the option to report to a Ministry or Department of Health.

6. ***Reviewer Feedback:*** *Add More Detail on Analytical Methods (Lines 143–144)*

*“Lines 143–144 state that descriptive statistics were calculated using Python 3.8.3, but there is no detail about the Packages used, handling of missing data, outlier management, and approach to consistency checks across sites. To enhance reproducibility and transparency, more detail is needed in this section.”*

**Response:**

The Statistical Analysis section has been expanded to specify the Python packages used, describe handling of missing data, and clarify why no outlier exclusion was performed.

7. ***Reviewer Feedback:*** *Device heterogeneity (Lines 114-118, 128-13)*

*“Include a supplementary table comparing device characteristics and justification for selection, as these differences could influence ease-of-use and accuracy because different HIV kits, glucometers, and BP machines were used across countries.”*

**Response:**

A new Figure (Figure 1) has been added comparing HIV self-test kits and blood glucose meters used across countries. This table is referenced in the Methods section with accompanying justification for their selection, which primarily was related to device regulation for self-testing and availability. We chose not to include the blood pressure devices as they were not used for self-testing and thus their self-testing regulatory status and device usability features were not being evaluated in this study.

8. ***Reviewer Feedback:*** *Generalizability of target health conditions*

*“The choice of health conditions (HIV, elevated blood pressure, altered glucose levels) reflects major public health concerns in the study sites. However, generalizability to other contexts, where disease burdens differ, may be different. This has implications for the claims of healthcare organization or cost reduction (e.g., line 61). If diseases with high prevalence drive test uptake, feasibility or relevance, extrapolation should be more cautiously stated. Self-testing for blood pressure requires access to medical devices. The manuscript should discuss the logistical feasibility of device distribution and maintenance outside clinic settings. Especially when advocating for ‘self-testing’.”*

**Response:**

We have revised the Introduction and Discussion sections, noting that feasibility and uptake may be influenced by local disease burden and may not extrapolate to settings with different epidemiologic profiles. We also added text addressing the logistical considerations of distributing and maintaining devices outside clinic settings.

9. ***Reviewer Feedback:*** *Safe testing discussion*

*“Without clinical oversight, self-testing may risk over-diagnosis, inappropriate repeat testing, or misinterpretation. The manuscript should address how the App, or any other measure in place, could prevent unsafe testing behaviors, and whether it can bear any legal or clinical responsibility. Over-diagnosis also undermines the potential for reduced healthcare usage given the resource constraints.”*

**Response:**

We have expanded the Discussion to address potential risks associated with self-testing, including misinterpretation. The revised text clarifies the role of in-app guidance, referral pathways, and confirmatory testing recommendations.

10. ***Reviewer Feedback:*** *Clarifying self-testing protocol*

*“Although the manuscript promotes self-testing, many tests were assisted by study staff. This undermines the feasibility claims for unassisted self-testing and may inflate acceptability, confidence, and usability outcomes.” AND “Confidence in re-using oral swabs or glucose finger-prick tests in the future cannot be extrapolated to the general population without acknowledging that participants gained confidence through guided first-use.”*

**Response:**

We have clarified throughout the Methods and Discussion sections that research team members were not permitted to assist participants who were self-testing. Participants were required to conduct the self-tests using only app-based instructions. In cases when participants did not want to conduct the self-tests on their own, they did **not** rate the usability of conducting the tests and were only asked to rate the usability of understanding the test instructions. We have made explicit clarifications about this process in the Methods section.

11. ***Reviewer Feedback:*** *Occupation*

*“The occupation categories appear problematic. For example, 52% of Kenyan participants are categorized as “other” and 72% as unemployed in South Africa, despite separate categories for housewives and students. How do these people manage to survive? These numbers warrant clarification.”*

**Response:**

We have used documented free text responses to the “Other” category and recategorized participants’ occupations. A detailed breakdown of this recategorization can be found in the public dataset published on the Harvard Dataverse (available at request of the authors due to the sensitive nature of the data). In the case of the high unemployment rate in South Africa, we do not have additional free text responses to clarify if these participants engage in informal or part-time work. However, in Phase II of this study, which is underway now, similar rates of unemployment have been found among different households in the same community that was surveyed.

12. ***Reviewer Feedback:*** *HIV self-testing eligibility*

*“A striking 53% declined HIV self-testing. Thus, the finding that 93% were willing to share results with the Ministry of Health pertains only to a subset, potentially biased toward those less concerned about privacy or stigma.”*

**Response:**

We have revised the Discussion to clarify that willingness to share HIV self-test results with the Ministry of Health reflects only participants who elected to test and does not represent the full study population. This limitation is now explicitly acknowledged, including the potential for selection bias related to privacy concerns or stigma.

Please let me know if you require any additional changes.

Thank you in advance,

Carolyn Oliver
