# Supplementary material for: Acceptability and usability of home-based HIV and blood glucose self-testing and home-based blood pressure measurement in Kenya, South Africa, and Zambia
Source: PLOS Glob Public Health. 2026 Sep 25;6(9):e0005445. doi: 10.1371/journal.pgph.0005445 (PMC13614574; doi:10.1371/journal.pgph.0005445)
Supplement: Supplementary file 5 [file pgph.0005445.s005.docx]

REVIEWER COMMENTS

Dear Authors,

This manuscript addresses an important and timely topic: the feasibility and acceptability of home-based self-testing for HIV, diabetes, and hypertension across three sub-Saharan African countries. The multicounty design, inclusion of both communicable and non-communicable diseases, and use of digital tools strengthen its potential contribution. However, several methodological inconsistencies and analytic limitations must be addressed before the conclusions can be considered fully supported by the data.

Strengths

Multicounty design, which demonstrated high participant recruitment across three diverse settings (N= 526), the use of a mobile app-assisted self-testing tool enhances relevance to digital health interventions. The study was ethically conducted with details of IRB approvals from all participating countries and a sufficient description of procedures and device types.

Concerns

1. Zambia did NOT conduct true HIV self-testing (Lines 118 – 122), this is a critical methodological issue, as this is a primary outcome of the study. Participants only self-interpreted the results; they did not self-administer the test. Despite this deviation, Zambia’s results are included in pooled analyses of “ease,” “confidence,” and “feasibility.”

This undermines the conclusions made as it inflates the usability metrics, biases pooled results upwards and makes acceptability comparisons across sites invalid. It basically challenges the main conclusion of the study that “home-based self-testing for HIV… was feasible across all sites.”

- It is important to conduct sensitivity analyses excluding Zambia’s HIV findings, present country-stratified feasibility results, revise the conclusions made, and mainly clarify whether the aim was *self-testing* or *home testing facilitated by staff*.

2. Hypertension was not self-tested (Lines 128 n-130), the title and conclusion suggest self-testing of all three conditions, but BP measurements were performed by research staff, not participants. Provide an explicit justification that BP self-testing was excluded due to protocol, device complexity, or concerns about measurement validity. Revise the title and abstract to reflect that **hypertension was measured at home by staff**, not self-tested, and clarify in the Methods and Discussion how this affects comparability across disease conditions.
3. At *Lines 134–141*, the manuscript describes differing sampling approaches: **Convenience sampling** in Kenya and Zambia, and **Kish grid random selection** in South Africa. These methodological inconsistencies affect internal validity and cross-country comparability, yet their implications are minimally discussed. Please expand the limitations section to explicitly address how non-probability sampling may bias feasibility estimates and prevalence comparisons.
4. Interpretations of Clinical Measurements

In your methods, you clearly state that Blood glucose tests were conducted **without fasting** (Line 221–222) and blood pressure was taken at a **single time point** (Line 286–287); however, in the Results section, the values are presented alongside **national prevalence estimates** (Table 3), which could mislead readers into interpreting your findings as prevalence data. Although the Discussion acknowledges this limitation, it is important to make clear throughout that readings are point in time, not diagnostic, and cannot be compared directly to national prevalence.

5. Feasibility and Usability Findings May Be Influenced by Bias (Lines 165–167; 236–243)

The manuscript reports exceptionally high ease-of-use ratings (≥90%) across all tests (Lines 165–167; 236–243). While encouraging, these findings may reflect: **Social desirability bias** during household visits, the **Hawthorne effect** due to the presence of research staff, and variation in test type availability across sites.

It is important to expand the Limitations section to discuss how these biases could inflate feasibility ratings.

6. **Add More Detail on Analytical Methods (Lines 143–144)**

Lines 143–144 state that descriptive statistics were calculated using Python 3.8.3, but there is no detail about the Packages used, handling of missing data, outlier management, and approach to consistency checks across sites. To enhance reproducibility and transparency, more detail is needed in this section.

7. Device heterogeneity (Lines 114-118, 128-13)

Include a supplementary table comparing device characteristics and justification for selection, as these differences could influence ease-of-use and accuracy because different HIV kits, glucometers, and BP machines were used across countries.

Conclusively, this is an important and relevant study; however, there is still conceptual confusion between self-administered testing, home-based testing conducted by research staff, and self-interpretation using a mobile app. To strengthen the scientific rigor of this paper, these should be clearly defined. The conclusion should also be strengthened as it currently overstates the findings, given the methodological inconsistencies. More caution should be exercised, reflecting the differences between self-testing, sampling methods, device variability, and country-level variations. With major revisions, this paper has the potential to contribute meaningfully to self-testing literature in LMICs.
