# Supplementary material for: Acceptability and usability of home-based HIV and blood glucose self-testing and home-based blood pressure measurement in Kenya, South Africa, and Zambia
Source: PLOS Glob Public Health. 2026 Sep 25;6(9):e0005445. doi: 10.1371/journal.pgph.0005445 (PMC13614574; doi:10.1371/journal.pgph.0005445)
Supplement: Supplementary file 6 [file pgph.0005445.s008.docx]

July 27, 2026

Manuscript reference number: PGPH-D-25-03165R1

Title: “Acceptability and Usability of Home-based HIV and Blood Glucose Self-Testing and Home-based Blood Pressure Measurement in Kenya, South Africa, and Zambia”

Dear PLOS Global Public Health Editor,

Thank you for providing comments and inviting us to resubmit a revised version of our paper. We have provided a point-by-point response to each piece of feedback raised by the editorial review below. A version of the updated manuscript with Track Changes has been provided. We have also submitted a clean version of the revised manuscript without the use of Track Changes.

All authors have contributed significantly to the work, have seen and approved of this manuscript, and can attest to the validity and honesty of the results. The contents of this article have not been published and the paper is not under review elsewhere for publication. Thank you for your consideration.

Please direct all correspondence to:

Carolyn R Oliver, MS

325 Ninth Ave; HMC# 359927; Seattle, WA 98104

Tel: +1 214-300-1777;

E-mail: [croliver@uw.edu](mailto:croliver@uw.edu)

Sincerely,

**Carolyn R Oliver, MS**

*Medical Student*

*University of Washington School of Medicine*

Thank you for allowing us to make revisions that strengthen the content and fit within the journal's style. Below is a summary of the changes made, based on your comments received via email from PLOS Global Public Health.

1. “The central concern is that the manuscript claims to assess feasibility but does not operationalise feasibility in implementation-science terms. Feasibility is inferred mainly from test uptake, self-reported ease of use, confidence in interpretation, and preference for home-based testing. These measures support a limited conclusion about acceptability and usability among participants who engaged with the study, but they do not establish feasibility of an implementable programme. Thant is, the authors appear to define feasilibity simply as being able to roll out the testing program. Point-of-care tests for HIV and glucose are already widely available in many LMIC settings, so the difficult question is not whether a rapid test can be performed once under study conditions. The relevant feasibility question concerns implementation: supply chains, regulatory approval for self-use, device/app provisioning, quality assurance, staff time, training burden, household workflow, privacy, cost per completed test, failure modes, referral processes, and linkage to care, to name a few off the top of my head. The manuscript should define feasibility using a recognised framework, such as Bowen feasibility domains or Proctor implementation outcomes, and report concrete process measures rather than treating diagnostic yield as evidence of feasibility.”

**Response:** We thank the reviewer for this important observation and agree with the underlying critique. We recognize that our data — test uptake, self-reported ease of use, confidence in interpretation, and preference for home-based testing — speak to usability and acceptability of the testing approach among participants who engaged with the study, not to the feasibility of implementing a home-testing program at scale (e.g., supply chains, regulatory pathways, provisioning, quality assurance, training burden, cost per completed test, and linkage to care). We have revised the manuscript title and text, replacing references to "feasibility" throughout the manuscript with "usability and acceptability," which more precisely characterizes our study's scope and have defined how we assessed each of these more clearly in the Methods section. We now note evaluating the feasibility of self-testing home-based programs as an area for future research in the Discussion.

2. “The manuscript should include a full implementation cascade for each testing modality. Current denominators shift between total enrolled participants, eligible participants, those offered testing, those who opted in, those who completed testing, and those who answered usability questions. This creates a risk of overstating feasibility by conditioning interpretation on those who successfully completed testing and then provided favourable ratings. For each modality, the authors should report the number approached, eligible, enrolled, offered testing, accepted, attempted, completed, obtained a valid result, interpreted correctly, referred, and linked to follow-up care. The preferred visual overview is a Sankey plot. Missingness should also be separated into meaningful operational categories: not asked, not applicable, declined, unable to complete, technical failure, stockout, staff error, and true "prefer not to answer." In a feasibility study, these are not nuisance categories. Rather, they are part of the outcome set of interest.”

Response: We agree that a clearer implementation cascade is needed to avoid overstating usability and acceptability by conditioning interpretation on the subset of participants who completed testing and provided favorable ratings. To address this, we have added a Sankey diagram (Figure 2) for each testing modality (HIV, blood glucose, and blood pressure), illustrating participant flow from enrolled, offered testing, accepted testing, manner of testing (self-conducted vs. staff-conducted), provided usability survey responses, test result, and referral to follow-up care. Supplemental Tables 1 and 2 provide information on HIV and blood glucose testing including the Ns by study site for completing a test. Referral to care was provided to all participants with a new positive HIV test, high blood glucose, or high blood pressure and this has now been noted in the Methods section. We have also revised the Methods section to accurately describe the missingness categories used (e.g., not asked, not applicable, declined, unable to complete, technical failure, stockout, staff error, and prefer not to answer). These categories were already applied and reported in the Results section; the Methods section has been updated to reflect this level of detail rather than the simplified description previously provided, ensuring consistency between the two sections.

3. “The cross-site comparability problem is substantial. The manuscript pools or summarises results across Kenya, South Africa, and Zambia, but the intervention differed materially by site. Kenya used the full app with participant-captured photos, South Africa and Zambia used a prototype with staff-captured photos, and Zambia did not implement true HIV self-testing because staff collected and applied the specimen. Blood pressure was also staff-measured rather than self-measured. Therefore, "feasibility of self-testing" is not a common construct across all sites. The primary presentation should be site-specific, with pooled results used only as descriptive context where the procedure was implemented comparably. The Zambia HIV arm should not contribute to claims about HIV self-testing feasibility. The manuscript also needs a Zambia-specific contextual section explaining why HIV self-testing was not implemented there, including regulatory approval, test availability, procurement constraints, prior community familiarity with finger-prick testing, and what would need to change for true self-testing implementation.”

Response:

We appreciate the reviewer's concern regarding cross-site comparability. Our intent was not to compare sites directly, but to characterize usability and acceptability of home-based testing across a broader, more representative range of sub-Saharan African settings than a single-site study could offer. We have added language to the Methods and Discussion clarifying that our pooled analysis is intended as a descriptive characterization across varied implementation contexts rather than a formal comparative analysis, and we have retained our summary figure (Figure 3) on this basis.

Regarding Zambia, our manuscript already notes that participants did not collect their own specimen due to OraQuick stockout, and that a study team member instead collected the specimen and applied it to a health-worker-use test. We also note that Zambia participants were not surveyed on HIV self-test usability, only on ease of understanding the app instructions, so Zambia data do not contribute to HIV self-testing usability claims. We have clarified language in the Methods and Discussion section to ensure these points are clearly communicated.

4. “The diagnostic testing results are over-emphasised relative to the feasibility question. HIV positivity, high non-fasting glucose, and high blood pressure readings are reported prominently, and the manuscript juxtaposes these values with national prevalence estimates. This is not a valid epidemiological comparison because the sample is not population-representative, sampling strategies differed across countries, glucose was non-fasting, blood pressure was measured once, and the age/sex structure was not standardised. These results should be described as screen-positive findings in a selected feasibility sample, not as prevalence estimates or evidence of representativeness. If retained, comparisons with national estimates should be clearly labelled as contextual background only.”

Response:

We agree that our screen-positive findings should not be interpreted as prevalence estimates, and we appreciate the opportunity to clarify this further. While our manuscript already included sentences after each comparison distinguishing our screening-based results from diagnosis-based national estimates, we recognize this distinction warranted more prominent framing. We have added an introductory caveat at the start of this section in the Discussion, clearly stating that our screen-positive findings for HIV, high blood glucose, and high blood pressure cannot be directly compared to national estimates of HIV seropositivity, diabetes, and hypertension, given that our home-based tests are screening tools requiring diagnostic confirmation, and that national estimates are presented in Table 3 solely to contextualize disease burden and the potential need for point-of-care testing, not as a formal epidemiological comparison.

5. “The study also needs a logic model. The manuscript claims that home-based self-testing could expand access and support public health surveillance, but it does not articulate the causal pathway linking inputs and activities to implementation outputs and health outcomes. A logic model should connect test kits, app/device, household visits, non-assistance rules, result capture, counselling/referral, and data sharing to outputs such as uptake, completion, valid results, time-on-task, support needs, and failures; then to short-term outcomes such as confirmatory testing, linkage to care, repeat testing, and behaviour change; and finally to longer-term impacts such as earlier diagnosis, improved control, and improved surveillance signal quality. Each reported measure should be mapped to a node in this logic model, with unmeasured but essential nodes explicitly identified for future work.”

Response:

We appreciate the reviewer's suggestion and agree that a full logic model would be a valuable undertaking, more appropriately suited to a dedicated modeling paper than the scope of the present usability and acceptability study. We still feel it is appropriate to note that participants' high willingness to share results with community health workers and Ministries/Departments of Health signals an opportunity for future research to explore self-testing public health surveillance programs in LMICs. We view the development of a formal logic model as a promising direction for future work and thank the reviewer for raising it.

6. “The statistical methods are simple and broadly acceptable for a descriptive feasibility study, but the reporting is incomplete. Formal hypothesis testing is unnecessary, but key descriptive proportions should still be presented with uncertainty intervals, especially by site. Participants are also nested within households, so any comparative summaries or confidence intervals should account for household clustering where relevant. The authors should additionally report household-level feasibility metrics because privacy, space, device familiarity, and household attitudes toward testing may affect more than one participant within the same household.”

Response:

We agree that participants nested within households may not be fully independent, and we thank the reviewer for raising this. As our key descriptive outcomes are reported as simple proportions rather than composite scores, and the majority (usability ratings) demonstrate a strong ceiling effect (>95% affirmative across nearly all items and sites), we do not believe household clustering meaningfully affects the precision of these estimates. However, for our reported preference for future location of self-testing, which shows greater variability across sites (82-95% reporting a preference for home-based self-testing), we fit a household-clustered GEE logistic model to generate 95% confidence intervals accounting for within-household correlation, yielding clustered estimates for home-based preference of 85% (95% CI: 79–90%) in Kenya, 95% (95% CI: 91–98%) in South Africa, and 81% (95% CI: 74–87%) in Zambia, with an overall preference estimate of 87% (95% CI 84-90%). We have added these clustered estimates to the Results section.

7. “The manuscript does not provide evidence of compliance with standard reporting guidelines for cross-sectional observational studies or feasibility/implementation studies. At minimum, a STROBE checklist and an appropriate feasibility/pilot or implementation reporting checklist should be provided. Guideline adherence would likely force reporting of core elements currently absent, including recruitment flow, denominators, missingness categories, site-specific implementation deviations, and operational constraints.”

Response:

We agree that formal documentation of adherence to reporting guidelines strengthens the manuscript. We have completed a STROBE checklist for cross-sectional studies and are submitting it as a supplementary file alongside the revised manuscript.

8. “The manuscript makes broad feasibility and programme-promise claims (including multi-disease self-testing and potential public health surveillance value), but it does not adequately benchmark its results against comparable studies in other jurisdictions, other diseases, or other target populations, nor does it explain how this study's implementation conditions differ from (or replicate) real-world programme delivery. The central contribution of a feasibility paper is not the existence of usability in one cohort under study support. Rather, it's importance is in understanding how the observed uptake/usability compares with known ranges from HIV self-testing distribution programmes (community-based, secondary distribution, unsupervised home use), with NCD self-monitoring/self-testing initiatives (glucose SMBG programmes; BP monitoring), and with digital app–supported diagnostics in similar settings. The manuscript should add a structured comparative discussion that (i) summarises what prior studies show on uptake, correct-use/invalid rates, support needs, and linkage-to-care across settings and populations, (ii) highlights what is novel here, and (iii) explains which aspects are likely transferable and which are study-specific. Without that comparative framing, readers cannot judge whether the reported ">90% easy" ratings are exceptional, expected, or artefacts of the study design.”

Response:

We agree that benchmarking our findings against comparable studies strengthens the manuscript's contribution. As we are reframing our study around usability and acceptability rather than feasibility (see response to Point 1), the reviewer's concern regarding feasibility benchmarking is addressed by this reframing. We have expanded our Discussion to more explicitly situate our findings relative to prior HIV self-testing and SMBG literature across uptake, correct interpretation, and support needs, and to clarify what is novel about our multi-disease, multi-country design versus what is consistent with prior single-disease or single-country studies. We have also added language distinguishing which of our findings (e.g., device and instruction usability) are likely transferable across similar LMIC settings, versus other site-specific obstacles that should be seriously considered before designing home-based, self-testing programs (e.g., stockouts, equipment familiarity).

9. “To be clear, the Discussion should situate the results more effectively against prior evidence. The manuscript should compare uptake, completion, invalid/indeterminate rates, support needs, confidence, willingness to share results, and linkage-to-care with studies in other jurisdictions, other disease conditions, and other populations, including HIV self-testing programmes, NCD self-monitoring initiatives, blood glucose self-monitoring, blood pressure home monitoring, and app-supported diagnostic programmes. Without this comparative framing, readers cannot judge whether the reported high ease-of-use ratings and home-testing preference are exceptional, expected, or artefacts of the researcher-supported study setting.”

Response:

We have expanded our Discussion to compare uptake, confidence, and linkage-to-care with prior HIV self-testing studies, and completion rates with prior SMBG literature in similar settings. We also note that while prior HIV self-testing studies have examined willingness to disclose results to family members or partners, we did not identify comparable literature examining willingness to share results with community health workers or government health authorities, suggesting this is a novel contribution rather than a finding we can directly benchmark.

Please let me know if you require any additional changes.

Thank you in advance,

Carolyn Oliver
