# Supplementary material for: Acceptability and usability of home-based HIV and blood glucose self-testing and home-based blood pressure measurement in Kenya, South Africa, and Zambia
Source: PLOS Glob Public Health. 2026 Sep 25;6(9):e0005445. doi: 10.1371/journal.pgph.0005445 (PMC13614574; doi:10.1371/journal.pgph.0005445)
Supplement: Peer review history

# Peer review: Decision Letter 0

**DOI:** 10.1371/journal.pgph.0005445.r001

**Authors:** Joel Francis

3 Dec 2025

PGPH-D-25-03165

Feasibility and Preferences for Home-based Self-Testing for HIV, Diabetes, and Hypertension in Kenya, South Africa, and Zambia

PLOS Global Public Health

Dear Dr. Oliver,

Thank you for submitting your manuscript to PLOS Global Public Health. After careful consideration, we feel that it has merit but does not fully meet PLOS Global Public Health’s publication criteria as it currently stands. Therefore, we invite you to submit a revised version of the manuscript that addresses the points raised during the review process.

Please submit your revised manuscript by Jan 17 2026 11:59PM. If you will need more time than this to complete your revisions, please reply to this message or contact the journal office at globalpubhealth@plos.org. When you're ready to submit your revision, log on to https://www.editorialmanager.com/pgph/ and select the 'Submissions Needing Revision' folder to locate your manuscript file.

Please include the following items when submitting your revised manuscript:

- A rebuttal letter that responds to each point raised by the editor and reviewer(s). You should upload this letter as a separate file labeled 'Response to Reviewers'.
- A marked-up copy of your manuscript that highlights changes made to the original version. You should upload this as a separate file labeled 'Revised Manuscript with Track Changes'.
- An unmarked version of your revised paper without tracked changes. You should upload this as a separate file labeled 'Manuscript'.

Guidelines for resubmitting your figure files are available below the reviewer comments at the end of this letter.

We look forward to receiving your revised manuscript.

Kind regards,

Joel Msafiri Francis, MD, MS, PhD

Academic Editor

PLOS Global Public Health

Journal Requirements:

1. Please include a complete copy of PLOS’ questionnaire on inclusivity in global research in your revised manuscript. Our policy for research in this area aims to improve transparency in the reporting of research performed outside of researchers’ own country or community. The policy applies to researchers who have travelled to a different country to conduct research, research with Indigenous populations or their lands, and research on cultural artefacts. The questionnaire can also be requested at the journal’s discretion for any other submissions, even if these conditions are not met.  Please find more information on the policy and a link to download a blank copy of the questionnaire here: https://journals.plos.org/globalpublichealth/s/best-practices-in-research-reporting. Please upload a completed version of your questionnaire as Supporting Information when you resubmit your manuscript.

2. Please amend your detailed Financial Disclosure statement. This is published with the article. It must therefore be completed in full sentences and contain the exact wording you wish to be published.

1. Please clarify all sources of funding (financial or material support) for your study. List the grants (with grant number) or organizations (with url) that supported your study, including funding received from your institution.

2. State the initials, alongside each funding source, of each author to receive each grant.

3. State what role the funders took in the study. If the funders had no role in your study, please state: “The funders had no role in study design, data collection and analysis, decision to publish, or preparation of the manuscript.”

4. If any authors received a salary from any of your funders, please state which authors and which funders.

3. Please insert an Ethics Statement at the beginning of your Methods section, under a subheading 'Ethics Statement'. It must include:

1) The name(s) of the Institutional Review Board(s) or Ethics Committee(s)

2) The approval number(s), or a statement that approval was granted by the named board(s)

3) (for human participants/donors) - A statement that formal consent was obtained (must state whether verbal/written) OR the reason consent was not obtained (e.g. anonymity). NOTE: If child participants, the statement must declare that formal consent was obtained from the parent/guardian.

4. If the reviewer comments include a recommendation to cite specific previously published works, please review and evaluate these publications to determine whether they are relevant and should be cited. There is no requirement to cite these works unless the editor has indicated otherwise.

[Note: HTML markup is below. Please do not edit.]

Reviewers' comments:

Reviewer's Responses to Questions

**Comments to the Author**

1. Does this manuscript meet PLOS Global Public Health’s publication criteria?

Reviewer #1: Partly

Reviewer #2: Partly

**********

2. Has the statistical analysis been performed appropriately and rigorously?

Reviewer #1: No

Reviewer #2: Yes

**********

3. Have the authors made all data underlying the findings in their manuscript fully available (please refer to the Data Availability Statement at the start of the manuscript PDF file)?

The PLOS Data policy

Reviewer #1: Yes

Reviewer #2: No

**********

4. Is the manuscript presented in an intelligible fashion and written in standard English?

Reviewer #1: Yes

Reviewer #2: Yes

**********

Reviewer #1: This manuscript addresses an important question regarding the feasibility of home-based self-testing for HIV, diabetes, and hypertension across Kenya, South Africa, and Zambia. The multicountry design and digital tool integration are strengths, and the study was ethically conducted with clear procedural descriptions.

However, several methodological issues require attention. Most critically, Zambia did not perform true HIV self-testing (Lines 118–122). Participants did not collect their own finger-prick specimens, yet their data were included in pooled analyses of feasibility and ease-of-use. This inflates usability estimates and undermines the study’s main conclusion that HIV self-testing was feasible across all sites. Sensitivity analyses excluding Zambia and stratified country-level results are needed, and the conclusions should be revised accordingly.

Hypertension was not self-tested (Lines 128–130), though the title and conclusions imply that all three conditions were self-tested. The manuscript should clarify that BP was measured by staff, explain why self-testing was excluded, and adjust the title/abstract.

Sampling methods differed substantially across countries (Lines 134–141), introducing bias and limiting comparability. The limitations section should explicitly address this. Additionally, blood glucose and BP were single, non-fasting, point-in-time measurements but are compared with national prevalence estimates, which may mislead readers.

Very high usability ratings (≥90%) may reflect social desirability or observer bias (Lines 165–167; 236–243). Statistical methods (Lines 143–144) require more detail on packages, missing-data handling, and validation steps. Device heterogeneity across countries (Lines 114–118; 128–130) also warrants clearer justification.

Overall, the study is valuable but requires major revision to address inconsistencies between self-testing vs. staff-facilitated testing, sampling limitations, device variability, and analytic gaps.

Reviewer #2: This manuscript presents a large, ambitious feasibility study conducted among 300 households across three African countries. The study’s scope, evaluating rapid diagnostic tests for both communicable and non-communicable diseases (NCDs), is commendable and sets it apart from many feasibility studies focused on single-disease rapid tests. The combination of finger-prick blood tests and oral swab saliva tests across multiple sub-Saharan African contexts further strengthens the relevance and applicability of the work. I endorse the value and scale of this study, though clarifications, refinements, and interpretative cautions are warranted in my opinion.

The discussion states that single time-point blood pressure measurements were not diagnostic for hypertension, and that non-fasting blood glucose tests were not diagnostic of diabetes. This is valid. However, these limitations must be consistently reflected throughout the manuscript. References that imply these tests “screened for hypertension or diabetes” (including the abstract) should be revised to emphasize that the study measured blood pressure and blood sugar levels, not clinical diagnoses. This distinction is essential for accurate interpretation.

The choice of health conditions (HIV, elevated blood pressure, altered glucose levels) reflects major public health concerns in the study sites. However, generalizability to other contexts, where disease burdens differ, may be different. This has implications for the claims of healthcare organization or cost reduction (e.g., line 61). If diseases with high prevalence drive test uptake, feasibility or relevance, extrapolation should be more cautiously stated. Self-testing for blood pressure requires access to medical devices. The manuscript should discuss the logistical feasibility of device distribution and maintenance outside clinic settings. Especially when advocating for “self-testing”.

Without clinical oversight, self-testing may risk over-diagnosis, inappropriate repeat testing, or misinterpretation. The manuscript should address how the App, or any other measure in place, could prevent unsafe testing behaviors, and whether it can bear any legal or clinical responsibility. Over-diagnosis also undermines the potential for reduced healthcare usage given the resource constraints.

Aggregating “never tested before” with “tested >12 months ago” may be epidemiologically logical, but from psychological and societal perspectives, these groups seem distinct.

Although the manuscript promotes self-testing, many tests were assisted by study staff. This undermines the feasibility claims for unassisted self-testing and may inflate acceptability, confidence, and usability outcomes. The results could be potentially framed as demonstrating feasibility for mobile health teams rather than independent self-testing. Even so, some barriers to clinic-based testing (transport, stigma, wait times) seem successfully mitigated in the current setup.

The study mentions issues with clinic-based systems such as lost results, stockouts, and understaffing, but similar challenges could occur in self-testing programs. Test supply chains, data transmission quality, and technical failures should be acknowledged as equivalent risks.

The occupation categories appear problematic. For example, 52% of Kenyan participants are categorized as “other” and 72% as unemployed in South Africa, despite separate categories for housewives and students. How do these people manage to survive? These numbers warrant clarification.

Confidence in re-using oral swabs or glucose finger-prick tests in the future cannot be extrapolated to the general population without acknowledging that participants gained confidence through guided first-use. Confidence in unguided testing, relying solely on the app, may be lower.

The manuscript does not address the potentially reduced sensitivity of self-collected samples. False negatives, especially for HIV, pose risks to individuals and communities. A dedicated discussion seems necessary.

A striking 53% declined HIV self-testing. Thus, the finding that 93% were willing to share results with the Ministry of Health pertains only to a subset, potentially biased toward those less concerned about privacy or stigma. Does willingness to share reflect trust in government and are clinic-based HIV test results currently accessible to government databases? In addition, how does privacy matters and concerns differ across countries?

The differing hypertension prevalence estimates (12%, 29%, 40% vs. 25%, 45%, 25.9% in Kenya, South Africa, Zambia) require more explanation from the start. As given by the authors, blood glucose and blood pressure fluctuate throughout the day, and interpreting values without clinical guidance may be unsafe. The discussion should emphasize the need for integrated clinical support or grounded decision trees.

Minor comments

- The references included in Table 3 for the estimated national prevalences require some more information. The current notation of references included in Table 3 for the estimated national prevalences confusing with the reported (%) higher up in the table.

- The fractions in table 4 are making it hard to interpret at first sight. The denominators are clear from the top rows, which present the total numbers, in my opinion. And the total numbers also have a %, to present the fraction of the total study population.

- Showing estimated prevalence of each health issue in the study with the national reference in one table would enhance the comparability for the reader. Now these numbers are in different tables.

In conclusion, this study offers innovative and valuable insights into multi-disease rapid testing feasibility across African contexts. However, revisions seem necessary to improve clarity on diagnostic limitations, generalizability, safety considerations, participant autonomy, and consistency in reporting. Strengthening these areas would enhance the manuscript’s impact and scientific rigor.

**********

what does this mean?). If published, this will include your full peer review and any attached files.

**Do you want your identity to be public for this peer review?**  If you choose “no”, your identity will remain anonymous but your review may still be made public.

For information about this choice, including consent withdrawal, please see our Privacy Policy

Reviewer #1: **Yes:**  Dr. Faith Lazarous Aikaeli

Reviewer #2: No

**********

[NOTE: If reviewer comments were submitted as an attachment file, they will be attached to this email and accessible via the submission site. Please log into your account, locate the manuscript record, and check for the action link "View Attachments". If this link does not appear, there are no attachment files.]

Attachment Submitted filename: REVIEWER COMMENTS_SELFTESTING.docx

# Author comment: Author response to Decision Letter 1

**DOI:** 10.1371/journal.pgph.0005445.r002

4 Mar 2026

Attachment Submitted filename: Response to Reviewers.docx

# Peer review: Decision Letter 1

**DOI:** 10.1371/journal.pgph.0005445.r003

**Authors:** Helen Howard

25 May 2026

PGPH-D-25-03165R1

Feasibility and Preferences for Home-based HIV and Blood Glucose Self-Testing and Home-based Blood Pressure Measurement in Kenya, South Africa, and Zambia

PLOS Global Public Health

Dear Dr. Oliver,

Thank you for submitting your manuscript to PLOS Global Public Health. After careful consideration, we feel that it has merit but does not fully meet PLOS Global Public Health’s publication criteria as it currently stands. Therefore, we invite you to submit a revised version of the manuscript that addresses the points raised during the review process.

Please submit your revised manuscript by Jul 21 2026 11:59PM. If you will need more time than this to complete your revisions, please reply to this message or contact the journal office at globalpubhealth@plos.org. When you're ready to submit your revision, log on to https://www.editorialmanager.com/pgph/ and select the 'Submissions Needing Revision' folder to locate your manuscript file.

Please include the following items when submitting your revised manuscript:

- A letter that responds to each point raised by the editor and reviewer(s). You should upload this letter as a separate file labeled 'Response to Reviewers'.
- A marked-up copy of your manuscript that highlights changes made to the original version. You should upload this as a separate file labeled 'Revised Manuscript with Track Changes'.
- An unmarked version of your revised paper without tracked changes. You should upload this as a separate file labeled 'Manuscript'.

Guidelines for resubmitting your figure files are available below the reviewer comments at the end of this letter.

As the corresponding author, your ORCID iD is verified in the submission system and will appear in the published article. PLOS supports the use of ORCID, and we encourage all coauthors to register for an ORCID iD and use it as well. Please encourage your coauthors to verify their ORCID iD within the submission system before final acceptance, as unverified ORCID iDs will not appear in the published article. *Only*  the individual author can complete the verification step; PLOS staff *cannot*  verify ORCID iDs on behalf of authors.

We look forward to receiving your revised manuscript.

Kind regards,

Helen Howard

Staff Editor

PLOS Global Public Health

Journal Requirements:

If the reviewer comments include a recommendation to cite specific previously published works, please review and evaluate these publications to determine whether they are relevant and should be cited. There is no requirement to cite these works unless the editor has indicated otherwise.

Additional Editor Comments (if provided):

[Note: HTML markup is below. Please do not edit.]

Reviewers' comments:

Reviewer's Responses to Questions

**Comments to the Author**

Reviewer #3: (No Response)

**********

publication criteria?

Reviewer #3: Yes

**********

3. Has the statistical analysis been performed appropriately and rigorously?

Reviewer #3: No

**********

4. Have the authors made all data underlying the findings in their manuscript fully available (please refer to the Data Availability Statement at the start of the manuscript PDF file)?

The PLOS Data policy

Reviewer #3: Yes

**********

5. Is the manuscript presented in an intelligible fashion and written in standard English?

Reviewer #3: Yes

**********

Reviewer #3: Dear Editor,

Thank you for the opportunity to provide a review of Manuscript PGPH-D-25-03165R1 entitled "Feasibility and Preferences for Home-based HIV and Blood Glucose Self-Testing and Home-based Blood Pressure Measurement in Kenya, South Africa, and Zambia." My comments relate primarily to the adequacy of the implementation and reporting of epidemiologic and statistical procedures.

I note that the manuscript had undergone a previous round of peer review. My review included the original version, the reviewers' comments, the authors rejoinder, and the revised manuscript (including the version with changes tracked).

This is a cross-sectional, multi-country household survey with optional home-based rapid testing conducted in peri-urban and rural communities in Kenya, South Africa, and Zambia, with household visits between 31 May and 4 August 2023. The population is household members aged 15 years and older included in this analysis, selected via convenience sampling in Kenya and Zambia, and Kish-grid procedures in South Africa. The "intervention" is an opt-in offer to self-test for HIV and blood glucose using an app-guided process (Zambia had staff-administered HIV specimen collection), plus optional staff-measured blood pressure. Outcomes are framed as feasibility/usability/acceptability and preferences (ease of instructions, ease of completing tests, confidence interpreting HIV results, preferred testing location, willingness to share results), alongside the measured test results (HIV positivity, non-fasting glucose thresholds, single blood pressure reading thresholds). The statistical approach is descriptive summaries (percentages, means/SD, medians/IQR) without inferential powering, with missing values coded as "prefers not to answer" and no outlier exclusion.

The central concern is that the manuscript claims to assess feasibility but does not operationalise feasibility in implementation-science terms. Feasibility is inferred mainly from test uptake, self-reported ease of use, confidence in interpretation, and preference for home-based testing. These measures support a limited conclusion about acceptability and usability among participants who engaged with the study, but they do not establish feasibility of an implementable programme. Thant is, the authors appear to define feasilibity simply as being able to roll out the testing program. Point-of-care tests for HIV and glucose are already widely available in many LMIC settings, so the difficult question is not whether a rapid test can be performed once under study conditions. The relevant feasibility question concerns implementation: supply chains, regulatory approval for self-use, device/app provisioning, quality assurance, staff time, training burden, household workflow, privacy, cost per completed test, failure modes, referral processes, and linkage to care, to name a few off the top of my head. The manuscript should define feasibility using a recognised framework, such as Bowen feasibility domains or Proctor implementation outcomes, and report concrete process measures rather than treating diagnostic yield as evidence of feasibility.

The manuscript should include a full implementation cascade for each testing modality. Current denominators shift between total enrolled participants, eligible participants, those offered testing, those who opted in, those who completed testing, and those who answered usability questions. This creates a risk of overstating feasibility by conditioning interpretation on those who successfully completed testing and then provided favourable ratings. For each modality, the authors should report the number approached, eligible, enrolled, offered testing, accepted, attempted, completed, obtained a valid result, interpreted correctly, referred, and linked to follow-up care. The preferred visual overview is a Sankey plot. Missingness should also be separated into meaningful operational categories: not asked, not applicable, declined, unable to complete, technical failure, stockout, staff error, and true "prefer not to answer." In a feasibility study, these are not nuisance categories. Rather, they are part of the outcome set of interest.

The cross-site comparability problem is substantial. The manuscript pools or summarises results across Kenya, South Africa, and Zambia, but the intervention differed materially by site. Kenya used the full app with participant-captured photos, South Africa and Zambia used a prototype with staff-captured photos, and Zambia did not implement true HIV self-testing because staff collected and applied the specimen. Blood pressure was also staff-measured rather than self-measured. Therefore, "feasibility of self-testing" is not a common construct across all sites. The primary presentation should be site-specific, with pooled results used only as descriptive context where the procedure was implemented comparably. The Zambia HIV arm should not contribute to claims about HIV self-testing feasibility. The manuscript also needs a Zambia-specific contextual section explaining why HIV self-testing was not implemented there, including regulatory approval, test availability, procurement constraints, prior community familiarity with finger-prick testing, and what would need to change for true self-testing implementation.

The diagnostic testing results are over-emphasised relative to the feasibility question. HIV positivity, high non-fasting glucose, and high blood pressure readings are reported prominently, and the manuscript juxtaposes these values with national prevalence estimates. This is not a valid epidemiological comparison because the sample is not population-representative, sampling strategies differed across countries, glucose was non-fasting, blood pressure was measured once, and the age/sex structure was not standardised. These results should be described as screen-positive findings in a selected feasibility sample, not as prevalence estimates or evidence of representativeness. If retained, comparisons with national estimates should be clearly labelled as contextual background only.

The study also needs a logic model. The manuscript claims that home-based self-testing could expand access and support public health surveillance, but it does not articulate the causal pathway linking inputs and activities to implementation outputs and health outcomes. A logic model should connect test kits, app/device, household visits, non-assistance rules, result capture, counselling/referral, and data sharing to outputs such as uptake, completion, valid results, time-on-task, support needs, and failures; then to short-term outcomes such as confirmatory testing, linkage to care, repeat testing, and behaviour change; and finally to longer-term impacts such as earlier diagnosis, improved control, and improved surveillance signal quality. Each reported measure should be mapped to a node in this logic model, with unmeasured but essential nodes explicitly identified for future work.

The statistical methods are simple and broadly acceptable for a descriptive feasibility study, but the reporting is incomplete. Formal hypothesis testing is unnecessary, but key descriptive proportions should still be presented with uncertainty intervals, especially by site. Participants are also nested within households, so any comparative summaries or confidence intervals should account for household clustering where relevant. The authors should additionally report household-level feasibility metrics because privacy, space, device familiarity, and household attitudes toward testing may affect more than one participant within the same household.

The manuscript does not provide evidence of compliance with standard reporting guidelines for cross-sectional observational studies or feasibility/implementation studies. At minimum, a STROBE checklist and an appropriate feasibility/pilot or implementation reporting checklist should be provided. Guideline adherence would likely force reporting of core elements currently absent, including recruitment flow, denominators, missingness categories, site-specific implementation deviations, and operational constraints.

The manuscript makes broad feasibility and programme-promise claims (including multi-disease self-testing and potential public health surveillance value), but it does not adequately benchmark its results against comparable studies in other jurisdictions, other diseases, or other target populations, nor does it explain how this study's implementation conditions differ from (or replicate) real-world programme delivery. The central contribution of a feasibility paper is not the existence of usability in one cohort under study support. Rather, it's importance is in understanding how the observed uptake/usability compares with known ranges from HIV self-testing distribution programmes (community-based, secondary distribution, unsupervised home use), with NCD self-monitoring/self-testing initiatives (glucose SMBG programmes; BP monitoring), and with digital app–supported diagnostics in similar settings. The manuscript should add a structured comparative discussion that (i) summarises what prior studies show on uptake, correct-use/invalid rates, support needs, and linkage-to-care across settings and populations, (ii) highlights what is novel here, and (iii) explains which aspects are likely transferable and which are study-specific. Without that comparative framing, readers cannot judge whether the reported ">90% easy" ratings are exceptional, expected, or artefacts of the study design.

To be clear, the Discussion should situate the results more effectively against prior evidence. The manuscript should compare uptake, completion, invalid/indeterminate rates, support needs, confidence, willingness to share results, and linkage-to-care with studies in other jurisdictions, other disease conditions, and other populations, including HIV self-testing programmes, NCD self-monitoring initiatives, blood glucose self-monitoring, blood pressure home monitoring, and app-supported diagnostic programmes. Without this comparative framing, readers cannot judge whether the reported high ease-of-use ratings and home-testing preference are exceptional, expected, or artefacts of the researcher-supported study setting.

Overall, the manuscript would be more accurate if reframed as an acceptability and usability demonstration under researcher-supported household conditions, rather than a full feasibility evaluation of a scalable home-testing programme. To support the stronger feasibility claim, the authors need to define feasibility explicitly, report a full implementation cascade and process metrics, separate site-specific procedures, provide Zambia-specific context, distinguish screening yield from feasibility, and add a logic model with appropriate reporting guideline compliance.

Thank you.

**********

what does this mean?). If published, this will include your full peer review and any attached files.

**Do you want your identity to be public for this peer review?**  If you choose “no”, your identity will remain anonymous but your review may still be made public.

For information about this choice, including consent withdrawal, please see our Privacy Policy

Reviewer #3: No

**********

[NOTE: If reviewer comments were submitted as an attachment file, they will be attached to this email and accessible via the submission site. Please log into your account, locate the manuscript record, and check for the action link "View Attachments". If this link does not appear, there are no attachment files.]

# Author comment: Author response to Decision Letter 2

**DOI:** 10.1371/journal.pgph.0005445.r004

26 Jul 2026

Attachment Submitted filename: Reviewer Letter response_25072026.docx

# Peer review: Decision Letter 2

**DOI:** 10.1371/journal.pgph.0005445.r005

**Authors:** Julia Robinson

24 Aug 2026

Acceptability and Usability of Home-based HIV and Blood Glucose Self-Testing and Home-based Blood Pressure Measurement in Kenya, South Africa, and Zambia

PGPH-D-25-03165R2

Dear Ms. Oliver,

We are pleased to inform you that your manuscript 'Acceptability and Usability of Home-based HIV and Blood Glucose Self-Testing and Home-based Blood Pressure Measurement in Kenya, South Africa, and Zambia' has been provisionally accepted for publication in PLOS Global Public Health.

Before your manuscript can be formally accepted you will need to complete some formatting changes, which you will receive in a follow up email. A member of our team will be in touch with a set of requests.

Please note that your manuscript will not be scheduled for publication until you have made the required changes, so a swift response is appreciated.

IMPORTANT: The editorial review process is now complete. PLOS will only permit corrections to spelling, formatting or significant scientific errors from this point onwards. Requests for major changes, or any which affect the scientific understanding of your work, will cause delays to the publication date of your manuscript.

If your institution or institutions have a press office, please notify them about your upcoming paper to help maximize its impact. If they'll be preparing press materials, please inform our press team as soon as possible -- no later than 48 hours after receiving the formal acceptance. Your manuscript will remain under strict press embargo until 2 pm Eastern Time on the date of publication. For more information, please contact globalpubhealth@plos.org.

Thank you again for supporting Open Access publishing; we are looking forward to publishing your work in PLOS Global Public Health.

Best regards,

Julia Robinson

Executive Editor

PLOS Global Public Health

***********************************************************

Reviewer Comments (if any, and for reference):

Reviewer's Responses to Questions

**Comments to the Author**

Reviewer #3: All comments have been addressed

**********

publication criteria?

Reviewer #3: Yes

**********

3. Has the statistical analysis been performed appropriately and rigorously?

Reviewer #3: Yes

**********

4. Have the authors made all data underlying the findings in their manuscript fully available (please refer to the Data Availability Statement at the start of the manuscript PDF file)?

The PLOS Data policy

Reviewer #3: Yes

**********

5. Is the manuscript presented in an intelligible fashion and written in standard English?

Reviewer #3: Yes

**********

Reviewer #3: Dear Editor,

Thank you for the opportunity to review Manuscript PGPH-D-25-03165R2 entitled "Acceptability and Usability of Home-based HIV and Blood Glucose Self-Testing and Home-based Blood Pressure Measurement in Kenya, South Africa, and Zambia."

I note that the manuscript underwent two previous rounds of peer review.

I would like to thank the authors for their professional and thoughtful engagement with the my comments throughout the revision process. I was particularly pleased to see the inclusion of the Sankey plot to illustrate denominator drift, as I believe this substantially improves the transparency and interpretability of the participant flow.

I am satisfied that the concerns I raised during the previous rounds of review have been addressed appropriately. I have no further substantive comments or recommendations.

I commend the authors on the care with which they have revised the manuscript and thank them for their constructive engagement throughout the review process.

Thank you.

**********

what does this mean?). If published, this will include your full peer review and any attached files.

**Do you want your identity to be public for this peer review?**  If you choose “no”, your identity will remain anonymous but your review may still be made public.

For information about this choice, including consent withdrawal, please see our Privacy Policy

Reviewer #3: No

**********
